# Phylogenomics of the Andean Tetraploid Clade of the American Amaryllidaceae (Subfamily Amaryllidoideae): Unlocking a Polyploid Generic Radiation Abetted by Continental Geodynamics

**DOI:** 10.3389/fpls.2020.582422

**Published:** 2020-11-05

**Authors:** Alan W. Meerow, Elliot M. Gardner, Kyoko Nakamura

**Affiliations:** ^1^USDA-ARS-SHRS, National Clonal Germplasm Repository, Miami, FL, United States; ^2^Singapore Botanic Gardens, National Parks Board, Singapore, Singapore; ^3^Institute of Environment, Florida International University, Miami, FL, United States

**Keywords:** anchored hybrid enrichment, Andes, Asparagales, biogeography, geophyte, molecular systematics, monocotyledons, phylogenetics

## Abstract

One of the two major clades of the endemic American Amaryllidaceae subfam. Amaryllidoideae constitutes the tetraploid-derived (*n* = 23) Andean-centered tribes, most of which have 46 chromosomes. Despite progress in resolving phylogenetic relationships of the group with plastid and nrDNA, certain subclades were poorly resolved or weakly supported in those previous studies. Sequence capture using anchored hybrid enrichment was employed across 95 species of the clade along with five outgroups and generated sequences of 524 nuclear genes and a partial plastome. Maximum likelihood phylogenetic analyses were conducted on concatenated supermatrices, and coalescent-based species tree analyses were run on the gene trees, followed by hybridization network, age diversification and biogeographic analyses. The four tribes Clinantheae, Eucharideae, Eustephieae, and Hymenocallideae (sister to Clinantheae) are resolved in all analyses with > 90 and mostly 100% support, as are almost all genera within them. Nuclear gene supermatrix and species tree results were largely in concordance; however, some instances of cytonuclear discordance were evident. Hybridization network analysis identified significant reticulation in *Clinanthus*, *Hymenocallis*, *Stenomesson* and the subclade of Eucharideae comprising *Eucharis*, *Caliphruria*, and *Urceolina*. Our data support a previous treatment of the latter as a single genus, *Urceolina*, with the addition of *Eucrosia dodsonii*. Biogeographic analysis and penalized likelihood age estimation suggests an origin in the Cauca, Desert and Puna Neotropical bioprovinces for the complex in the mid-Oligocene, with more dispersals than vicariances in its history, but no extinctions. *Hymenocallis* represents the only instance of long-distance vicariance from the tropical Andean origin of its tribe Hymenocallideae. The absence of extinctions correlates with the lack of diversification rate shifts within the clade. The Eucharideae experienced a sudden lineage radiation ca. 10 Mya. We tie much of the divergences in the Andean-centered lineages to the rise of the Andes, and suggest that the Amotape—Huancabamba Zone functioned as both a corridor (dispersal) and a barrier to migration (vicariance). Several taxonomic changes are made. This is the largest DNA sequence data set to be applied within Amaryllidaceae to date.

## Introduction

Amaryllidaceae J.St.-Hil. is a family of herbaceous monocots with a cosmopolitan distribution containing approximately 90 genera and 1,700–1,800 species (Meerow and Snijman, 1998) that originated in Africa (Meerow et al., 1999). The family comprises three subfamilies (APG, 2003, 2009): Allidoideae Herb. (=Alliaceae J.G. Agardh, the economically-important onion family, 13 genera and 795 spp., the majority in *Allium* L.), South African Agapanthoideae Endl. (former Agapanthaceae F. Voight, one genus of ca. nine spp.), and Amaryllidoideae Burnett (Amaryllidaceae s.s., ca. 75 genera and ca. 900 spp.). The latter is the subject of this study and has a broad distribution, with a center of diversity in the southern hemisphere tropics. To this subfamily belong many of the world’s most celebrated ornamental bulbs, including daffodils (*Narcissus* L.), snowdrops (*Galanthus* L.) and “amaryllis” (*Hippeastrum* Herb.). Its species are characterized by 6-parted flowers with undifferentiated tepals and inferior ovaries, borne in pseudoumbels atop a leafless stalk known as a scape.

Three tribes of Amaryllidaceae subfam. Amaryllidoideae are almost entirely restricted to Africa (Amaryllideae Dumort., Cyrtantheae W. Aiton, and Haemantheae Hutch.) and one to Australasia (Calostemmateae D.Müll.-Doblies & U.Müll-Doblies) (Meerow and Snijman, 1998; Meerow et al., 1999). These tribes represent the first branches in published phylogenies of the subfamily (Meerow et al., 1999; Ito et al., 1999; Meerow, 2010; Rønsted et al., 2012). Perhaps the most unexpected resolution in early plastid DNA phylogenies of the family (Meerow et al., 1999; Ito et al., 1999), was the sister relationship of the endemic American clade of subfam. Amaryllidoideae to the Eurasian clade of the subfamily (tribes Galantheae Salisb., Lycorideae Traub, Narcisseae Endl., and Pancratieae Salisb.; Meerow et al., 2006). The dispersal of the Old World genus *Crinum* L. (tribe Amaryllideae) into the Americas is considered a separate event (Meerow et al., 2003; Kwembeya et al., 2007). This Eurasian/American clade relationship has led some to declare it as evidence of a boreotropical (Wolfe, 1975; Tiffney, 1985a,b; Tiffney and Manchester, 2001) origin for the American clade (Christenhusz and Chase, 2013), despite cladistic patterns that are inconsistent with expectations of the boreotropics hypothesis, an absence of fossils (Meerow et al., 2000), and a lack of biogeographic analyses.

The relationships of the endemic American genera were inferred using the internal transcribed spacer regions of nuclear ribosomal DNA (Meerow et al., 2000). These major relationships have also been supported by plastid data (Meerow et al., 1999, 2000; Meerow and Snijman, 2005; Meerow, 2010). The endemic American genera of the family were resolved as two major clades (Meerow et al., 1999, 2000). The “hippeastroid” clade are diploid (*x* = 11) or dysploid (*x* = 6, 8, 9), and primarily the extra-Andean element of the family, comprising the Brazilian endemic tribe Griffinieae Ravenna (*Cearanthes* Ravenna, *Griffinia* Ker-Gawl. and *Worsleya* Traub) (Campos-Rocha et al., 2019a,b), and the tribe Hippeastreae Herb. ex Sweet. (García et al., 2019). Several genera within the hippeastroid clade were recovered as polyphyletic (*Rhodophiala* C. Presl., *Zephyranthes* Herb.) with ITS (Meerow et al., 2000) and the possibility of reticulate evolution (i.e., early hybridization) in these lineages was hypothesized (Meerow, 2010; Meerow et al., 2000), and later confirmed with further analysis of plastome and multiple nuclear gene sequences (García et al., 2014, 2017). Hippeastreae constitutes two main clades, the subtribe Hippeastrinae Walp and the Chilean endemic subtribe Traubiinae D. & U. Müll.-Doblies (García et al., 2014, 2017, 2019). In contrast to the Hippeastrinae, the Traubiinae exhibits a mostly tree-like pattern of evolution (García et al., 2017). García et al. (2019) presented a new classification scheme for Hippeastreae that reflects its reticulate phylogeny.

**FIGURE 1:**
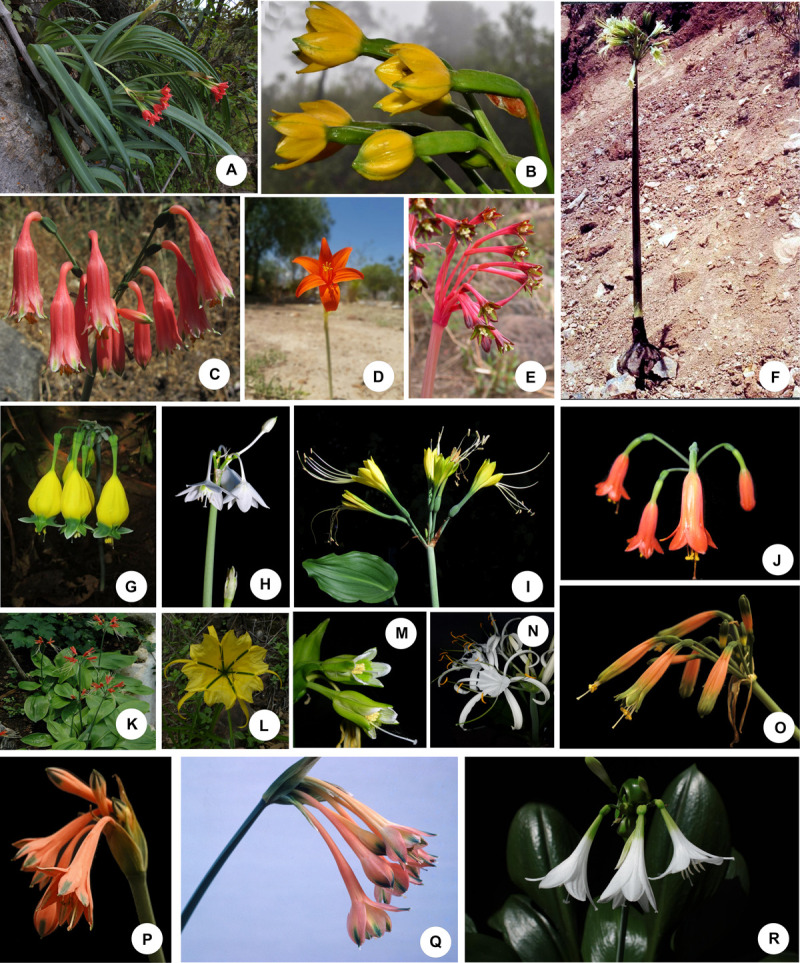
Representative species of the Andean tetraploid clade of the American Amaryllidaceae. **(A–C,P,Q)** Tribe Clinantheae. **(D,E)** Tribe Eustephieae. **(F–K,O,R)** Tribe Eucharideae. **(L–N)** Tribe Hymenocallideae. **(A)** *Paramongaia milagroantha* (Leiva & Meerow) Meerow. **(B)** *Pamianthe ecollis* Silverst., Meerow & Sánchez-Taborda. **(C)**. *Clinanthus campodensis* (Ravenna) Meerow. **(D)** *Pyrolirion tubiflorum* L’Hér.) M.Roem. **(E)** *Hieronymiella latifolia* (R.E.Fr.) Di Fulvio & Hunz. **(F)** *Rauhia albescens* Meerow & Sagást. **(G)** *Urceolina pendula* Herb. **(H)** *Eucharis cyaneosperma* Meerow. **(I)** *Eucrosia dodsonii* Meerow & Dehgan. **(J)** *Stenomesson ecuadorense* Meerow, Oleas & L.Jost. **(K)** *Eucrosia bicolor* Ker Gawl. **(L)** *Leptochiton helianthus* (Ravenna) Gereau & Meerow. **(M)** *Ismene parviflora* Meerow & A. Cano. **(N)** *Hymenocallis speciosa* (L.f. ex Salisb.) Salisb. **(O)** *Phaedranassa ventricosa* Baker. **(P)** *Clinanthus incarnatus* (Kunth) Meerow. **(Q)** *Clinanthus variegatus* (Ruiz & Pav.) Meerow. **(R)** *Caliphruria subedentata* Baker. Photo credits: **(A,C,L)** Segundo Leiva. **(B)** Jhon A. Sánchez-Taborda. **(D,I–K,N–R)** Alan Meerow. **(E)** John Wood. **(F)** Abundio Sagástegui. **(G,H)** Günter Gerlach. **(M)** Asuncíon Cano. Photos used by permission.

The second major clade in the Americas constitutes the tetraploid-derived (*n* = 23) Andean-centered tribes (Figure 1), most of which have 46 chromosomes (Meerow, 1987c). This somatic number may have arisen by duplication or fission of one chromosome in an ancestor with 2*n* = 22, considered ancestral in the subfamily (Meerow and Snijman, 1998), followed by genome doubling, which restored secondary balance (Satô, 1938; Bose, 1962). Many genera of the clade have a staminal corona or cup formed by the proximal connation of the filaments (Meerow and Snijman, 1998; Waters et al., 2013). The Andean clade is characterized by three consistent indels in nrDNA, two in the ITS1 and one in the ITS2 regions (Meerow et al., 2000).

Currently, there are 18 recognized genera in the clade, and ca. 200 spp. (Meerow and Snijman, 1998). Tribe Eustephieae Hutch., which is most diverse from southern Peru through Bolivia and Argentina, is the first branch of this clade, and lacks the indel in ITS2. The tribe Hymenocallideae (D. & U.Müll.-Doblies) Meerow (Meerow et al., 2002), which extends from the central Andes north to the southeastern United States (Figures 1L–N), and its exclusively Andean sister tribe Clinantheae Meerow (*Clinanthus* Herb., Figures 1C,P,Q; *Paramongaia* Velarde, Figure 1A; *Pamianthe* Stapf, Figure 1B) were recognized. *Clinanthus* was segregated from a polyphyletic *Stenomesson* Herb., which is now reserved for species of the genus with pseudopetiolate leaves (Meerow et al., 2000). A pseudopetiolate-leafed clade, containing elements of both Eucharideae Hutch. and Stenomesseae Traub (tribe Eucharideae, Figures 1F–K,O,R), was also resolved.

Despite this progress, several relationships within the Andean tetraploid clade were unresolved or weakly supported in these previous studies. Sequence capture using anchored hybrid enrichment, also known as Hyb-Seq (Cronn et al., 2012; Lemmon et al., 2012; Lemmon and Lemmon, 2013; Weitemier et al., 2014) has been informative at various taxonomic levels in plants (Fragoso-Martínez et al., 2017; Wanke et al., 2017; Carter et al., 2019; Granados Mendoza et al., 2020; Larridon et al., 2020; Murphy et al., 2020). The methodology yields sequences of hundreds of single or low copy nuclear loci.

In this paper, we present the results of such a phylogenomic approach across the Andean tetraploid clade of Amaryllidaceae subfam. Amaryllidoideae. We also analyze a partial plastome alignment of the clade, extracted and assembled from the sequence capture raw data, and from these two data matrices infer potential reticulation events, biogeographic patterns, and dates of divergence. We sought to test the following hypotheses: (1) phylogenomic data support the previous tribal and generic classification of the tetraploid Andean clade inferred from ITS and several plastid loci, (2) reticulation with each tribe is limited to interspecific gene flow at the generic level, (3) geological events in the Andean region were primary factors in the generic diversification of the clade, and (4) polyploidy and resulting paralogy are not necessary impediments to accurate phylogenomic inference. To date, this is the largest DNA sequence data set applied to Amaryllidaceae.

## Materials and Methods

### Sampling

We used genomic DNA from 100 species for the sequence capture, 95 from the Andean clade, with *Pancratium zeylanicum* L. (Eurasian clade) as outgroup, but also including four species of the hippeastroid clade (Supplementary Table 1). Every genus in the Andean clade was sampled, except for *Mathieua* Klotzch, presumed extinct and likely synonymous with *Stenomesson* (Meerow, 1987d). The most heavily sampled genus was *Hymenocallis* Salisb., with 42 samples. Approximate percent sampling of recognized spp. of each genus in our study is as follows: *Caliphruria*, 75%; *Chlidanthus* Herb., 25%; *Clinanthus*, 36%; *Eucharis*, 39%; *Eucrosia*, 71%; *Eustephia* Cav., 25%; *Hieronymiella* Pax, 17%; *Hymenocallis* Salisb., 59%; *Ismene* Salisb., 25%; *Leptochiton* Sealy, 50%; *Pamianthe*, 66%; *Paramongaia*, 100%; *Phaedranassa*, 44%; *Plagiolirion* Baker, 100%; *Pyrolirion* Herb., 50%; *Rauhia* Traub, 20%; *Stenomesson* Herb., 35%; *Urceolina* Rchb., 25%.

### Sequencing

We identified putative orthologous single-copy nuclear (SCN) loci from two floral transcriptomes (*Griffinia liboniana* C. Morren and *Rauhia staminosa* Ravenna) previously obtained through Beijing Genomics Institute (Shenzhen, China) using MarkerMiner 1.0 (Chamala et al., 2015) with a minimum sequence length of 300 bp and the SCN gene reference set of *Oryza sativa* L. curated by De Smet et al. (2013). MarkerMiner identified 751 SCN orthogroups which were reduced to 735 loci by excluding the orthogroups containing apparent paralogs, and sequence alignments were provided to Rapid Genomics (Gainesville, FL, United States) for probe design. A total of 45,054 probes of 120 bp were designed at 3× coverage across predicted exons of longer than 120 bp. DNA was extracted from 20 to 100 mg of dried or fresh leaf tissue using FastDNA Kit (MP Biomedicals, Irvine, CA, United States) and provided to Rapid Genomics with whom we contracted for sequence capture and initial bioinformatic analysis. DNA libraries were made by random mechanical shearing of our genomic DNA to an average size of 400 bp followed by an end-repair reaction, ligation of an adenine residue to the 3′-end of the blunt-end fragments to allow the ligation of barcoded adapters, and PCR-amplification of the library. SureSelect probes (Agilent Technologies, Santa Clara, CA, United States) were used for solution-based target enrichment of a pool of 16 libraries following the SureSelect^xt^ Target Enrichment System for Illumina Paired-End Multiplexed Sequencing Library protocol. Captured libraries were sequenced using Illumina HiSeq 3000 (Illumina, San Diego, CA, United States) to generate paired-end 100-bp reads.

### Plastome Assembly

Adapter sequences and base calls with a quality score of lower than 36 were trimmed from raw reads using Trimmomatic 0.36 (Bolger et al., 2014), and duplicate reads were removed with Dedupe software in the BBMap package v36.92 (Bushnell, 2014). Quality-filtered reads of *Clinanthus variegatus* (Ruiz & Pav.) Meerow were aligned against the plastid genome of *Hippeastrum cipoanum* (Ravenna) Meerow (García et al., 2017) using BWA 0.7.12-r1039 with the MEM algorithm (Li and Durbin, 2009) and other default parameters. The resulting alignment BAM file was imported, visually inspected, and manually corrected in Geneious 9.1.7 (Kearse et al., 2012), and the consensus sequence was extracted from the alignment. Quality-filtered reads of the other 99 species were initially aligned against the *C. variegatus* reference sequence using BWA, and a consensus sequence of each species was extracted in Geneious as described above. Then, the sequence reads of each species were aligned against the consensus sequence of its own species in the second round to fill gaps resulting from mismatches between the *C. variegatus* reference sequence and reads of other species. The mapping step was repeated until no more additional reads were aligned to the reference. Because the coverage of the small single copy region (SSC) of the plastomes was very low for most of the samples, our final matrices only include the large single copy region (LSC) and one of the inverted repeat regions (IRb). We manually trimmed difficult to align (mostly AT-rich) non-coding portions.

### Bioinformatics of Nuclear DNA

Rapid Genomics used a pipeline described in Breinholt et al. (2018) with a few modifications to process the capture data for phylogenetic analyses. Briefly, adaptors and bases with a Phred score (Ewing and Green, 1998; Ewing et al., 1998) below 20 were removed from raw Illumina reads using Trim Galore! ver. 0.4.0^1^ allowing a minimum read size of 30 bp. Filtered reads of each taxon highly similar to probe regions were selected and assembled *de novo* for each exon with iterative baited assembly (IBA) using the Python script IBA.py (Breinholt et al., 2018) with a minimum of 5× kmer coverage. To estimate copy numbers and orthology of captured sequences, assembled sequences were aligned with MAFFT 7.x (Katoh and Standley, 2013), trimmed to the probe region using extract_ probe_ region.py (Breinholt et al., 2018), and mapped to the *Phalaenopsis equestris* (Schauer) Rchb.f. genome using BLASTN (Camacho et al., 2009). The blast results were filtered with the scripts s_ hit_ checker.py and ortholog_filter.py (Breinholt et al., 2018), and those sequences that mapped to the same single location on the *P. equestris* genome were considered orthologs. The orthologous sequences including flanking regions were aligned with MAFFT, and loci that were represented by at least 70% of the sampled taxa were included in the final dataset. Difficult to align columns in flanking regions in the alignments were removed using the scripts alignment_DE_trim.py and flank_dropper.py (Breinholt et al., 2018).

### Reduction of Paralogs

Because the assembled loci contained considerable numbers of paralogs (Supplementary Figure 1), we used the tree-based pipeline described by Yang and Smith (2014) to generate sets of one-to-one orthologs and prune extraneous paralogs. First, we inferred a phylogenetic tree for each locus using FastTree2 (Price et al., 2010), or for the CDS-only data sets, RAxML 8.2.12 (Stamatakis, 2014). The scripts used to implement the Yang and Smith (2014) pipeline were downloaded from https://bitbucket.org/yangya/phylogenomic_dataset_construction/src/master/. Pruning then proceeded as follows: we used trim_tips.py to remove terminal tips with an absolute branch length of >0.5 substitutions/site. We then masked mono- and para- phyletic tips with the same sample name using mask_tips_by_taxonID_transcripts.py, thus removing alleles and extremely shallow (within individual) paralogs. Using cut_long_internal_branches.py, we cut deep paralogs based on an internal branch length of >0.5 substitutions/site. For final ortholog pruning, we used prune_paralogs_MO.py, retaining trees with at least 10 tips remaining. These pruned trees and sub-trees were used to write new fasta files with the write_fasta_files_from_trees.py script. The new locus files were re-aligned using MAFFT (Katoh and Standley, 2013). To compare this method with a paralog-agnostic approach, we also analyzed a supermatrix with RAxML version 8.2.12 (Stamatakis, 2014) using consensus sequences generated from all of the paralogs.

Several supermatrices were used for analysis, differing by the percentage of missing data. For each data set, the supermatrix was constructed using the fasta_merge.py script from HybPiper (Johnson et al., 2016). Supermatrices of coding regions only were also analyzed. Compilation and editing of all alignments was conducted with Geneious Prime 2020.0.5^2^.

### Data Analyses

#### Supermatrices

RAxML version 8.2.12 (Stamatakis, 2014) was used to conduct partitioned maximum likelihood (ML) best tree and thorough bootstrap analyses on the CIPRES Science Gateway (Miller et al., 2010), with an estimate of 25 per site rate categories, with joint branch length optimization, and the GTRCAT model. Three hundred bootstrap replicates were run, and a 50% majority rule bootstrap consensus tree was constructed using the SumTrees component of the package DendroPy v. 4.4.0 (Sukumaran and Holder, 2010). We manually created a “tanglegram” from the best ML 70% taxon coverage nuclear supermatrix and the plastome tree to highlight any cytonuclear discordance.

#### Species Tree Estimation

For each locus, a (ML) gene tree was inferred using RAxML under either the GTRCAT (for data sets with >50 samples) or GTRGAMMA (for data sets with <50 samples) models, with 100 rapid bootstrap replicates. Nodes with less than 30% bootstrap support were collapsed using SumTrees (Sukumaran and Holder, 2010). These collapsed trees were used to infer coalescent-based species trees with ASTRAL-III (Zhang et al., 2018). Node support was calculated using both local posterior probability (LPP, normalized quartet scores, representing gene tree concordance at each node) and bootstrap (100 replicates).

#### Species Network Analysis

SplitsTree v. 4.15.1 (Huson, 1998; Huson and Bryant, 2006) was used to infer reticulate evolution within the Andean tetraploid clade. Three separate analyses were conducted: Clinantheae, Eucharideae and the genus *Hymenocallis*. *Pyrolirion albicans* Herb. (Eustephieae) was included as outgroup in analyses of Clinantheae and Eucharideae; for *Hymenocallis*, *Leptochiton quitoensis* (Herb.) Sealy was designated as outgroup. The concatenated supermatrix of 70 nuclear gene alignments with 99% taxon coverage across our original 100 samples as analyzed (120 kbp). *Paramongaia viridiflora* (Ruiz & Pav.) Meerow was dropped from the Clinantheae alignment because only eight genes (of 70) were successfully recovered from this herbarium sample. We used the NeighborNet distance transformation with uncorrected *P* distance to draw equal-angle hybridization networks (Albrecht et al., 2012) with 300 bootstrap iterations, and also conducted phi tests for recombination (Bruen et al., 2006).

#### Dating and Diversification Rate Analyses

We estimated age divergence within the Andean clade using penalized likelihood estimation on the best 90% taxon coverage supermatrix tree. This was conducted with the chronos function in APE v.5.3 (Paradis and Schliep, 2019) in R v. 4.0 (R Core Team, 2017), with two different models, “relaxed” and “correlated” (Kim and Sanderson, 2008; Paradis, 2013). Two calibrations points were placed on the tree. We estimated lower and upper bounds of 30–33 Mya for the stem node of the American clade, based on an inferred estimate from the dated tree in Santos-Gally et al. (2012) for the most recent common ancestor (MRCA) of the Eurasian and American clades of Amaryllidaceae subfam. Amaryllidoideae, and 10–12 Mya for the stem node of *Rauhia* Traub, based on fossil-supported estimated age of seasonally dry inter-Andean forests in the central Andes (Caetano et al., 2008; Quintana et al., 2017a). *Rauhia* is endemic to the Marañon dry woodlands of northern Peru (Meerow and Nakamura, 2019). The smoothing parameter (λ) was chosen using the cross-validation method in the chronopl function (testing λ = 0 and 0–5), selecting the value of λ that minimized the cross-validation statistic (Sanderson, 2002). The resulting trees were visualized using the densiTree function in phangorn 2.4.0 (Schliep, 2011), and the time calibrated tree representing the central tendency of these analyses was selected. Because the cross-validation method (Sanderson, 2002) failed to detect a single preferred value for the smoothing parameter (λ), we ran the analysis with 21 values of λ, from 10^–5^ to 10^15^. The resulting trees were visualized using the densiTree function in phangorn 2.4.0 (Schliep, 2011), and because several had equally likelihood scores, the time calibrated tree representing the central tendency of these analyses was selected. Confidence intervals on node ages are not provided in this study since the penalized likelihood approach used does not address model or calibration placement and timing uncertainty. A geologic timescale based on the strat2012 dataset was added to the tree using PHYLOCH v 1.5-3 (Heibl, 2008). The best tree was visualized using FigTree v. 1.44 (Rambaut, 2019).

To determine whether there have been any diversification rate shifts in the Andean tetraploid clade, we performed a speciation-extinction analysis on the relaxed dated tree produced by APE with BAMM v 2.5.0 (Rabosky et al., 2013, 2014a; Rabosky, 2014) and the R program BAMMtools (Rabosky et al., 2014b). We ran 50 M Markov Chain Monte Carlo (MCMC) generations with four chains, using priors set by BAMMtools from our tree. We estimated the amount of missing data for each clade (usually = genus) in the tree, and set the expected number of rate shifts to one. Convergence of the runs and the number of sampled rate shifts were tested using effective sample sizes (ESS) with R package coda v. 0.19-3 (Plummer et al., 2006).

#### Biogeographic Analysis

We used BioGeoBears (Matzke, 2012, 2013, 2014) as implemented through RASP v. 4.2 (Yu et al., 2015) with R v.4.0 to conduct biogeographic analyses. The input tree was the best 90% taxon coverage supermatrix tree. Outgroups were stripped from the tree as recommended by the RASP authors. We first implemented the six model test to determine the most appropriate model for our data by the weighted AICc statistic, subsequently implementing the model with the highest score. The only consequence of using a single tree was that the S-DIVA method could neither be evaluated nor used. Fifteen singular areas were designated in the analyses derived from the Neotropical bioregions and provinces of Morrone (2014) and Antonelli et al. (2018)with the exception of the Nearctic Realm/Eastern North America Bioregion (Ricketts et al., 1999): A = Napo Province, B = Cauca Province, C = Puna Province, D = Desert Province, E = Páramo Province, F = Yungas Province, G = Ecuadorian Province, H = Eastern North America Bioregion (Nearctic Realm), I = Western Ecuador Province, J = Ucayali Province, K = Chocó-Darién Province, L = Prepuna Province, M = Mesoamerica Bioregion, N = Guianan Lowlands Province, and O = West Indies Bioregion. We limited areas at any node to three, and mapped only the most likely on the tree. We also similarly ran an analysis on the best ML tree found by RAxML for the partial plastome supermatrix.

## Results

### Sequence Alignments

The complete sequence supermatrix included all loci that survived the paralog pruning, and was 730,500 bp in length, comprising 524 genes (Supplementary Table 2). We also created a supermatrix of all genes that included sequences from 70% (260 genes, 277,173 bp), 90% (137 genes, 201,059 bp), and 99% (70 genes, 120,000 bp) of the taxa, respectively.

The plastome supermatrix was 122,549 bp in length. The LSC consisted of 94,938 bp; the IR was 27,611 bp. Genera of the tribe Eucharideae exhibited substantial re-organization and pseudogenization of the genes of the *ndh* family.

### Supermatrix ML Analyses

**FIGURE 2:**
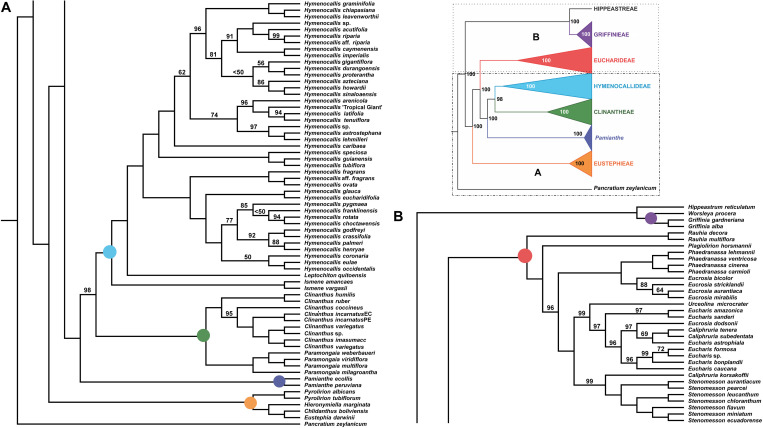
Best tree from maximum likelihood analysis of partitioned 524 nuclear gene supermatrix of the Andean tetraploid clade of Amaryllidaceae subfam. Amaryllidoideae, with bootstrap percentages <100 shown above branches (all unmarked branches have 100% BP). The tree is divided into two sections **(A,B)** for readability; refer to the colored inset (a cartoon of the tree collapsed to the tribal level) as a guide. Colored circles at the nodes in the full tree conform to clade colors in the inset.

All of the supermatrix analyses resolved a well-supported monophyletic Andean tetraploid clade (Figure 2) consisting of the four tribal subclades: Clinantheae, Eucharideae, Eustephieae, and Hymenocallideae, all with 100% bootstrap (BP) with a single exception in Clinantheae. The genus *Pamianthe* in the analyses with all genes (both coding + flanking regions) is sister to Clinantheae/Hymenocallideae (Figure 2,100% BP), but with coding regions alone (Supplementary Figure 2), it is the first branch of Clinantheae (94% BP). Trees from the 70 and 90% taxa coverage supermatrices (Supplementary Figures 3, 4) placed *Pamianthe* as the first branch in Clinantheae with 94 and 91% BP, respectively.

The tribe Eustephieae was sister to rest of the Andean clade, within which *Pyrolirion* Herb. was the first branch, followed by *Hieronymiella* Pax, then a sister relationship between *Chlidanthus* Herb. and *Eustephia* Cav. This topology occurred in all of our trees, as well as the plastome analysis, with 100% BP at each node. Clinantheae (less *Pamianthe*) and Hymenocallideae were sister tribes with 98% BP. Together with *Pamianthe*, they formed the sister clade to Eucharideae. The *Paramongaia* subclade of Clinantheae was supported with 100% BP by all supermatrices. *Clinanthus* formed two subclades. The larger of the two represented *Clinanthus incarnatus* (Kunth) Meerow, *C. variegatus* and their variants. *Clinanthus coccineus* (Ruiz & Pav.) Meerow was the first branch. *C. incarnatus* was monophyletic and distinct from *C. variegatus*.

**FIGURE 3:**
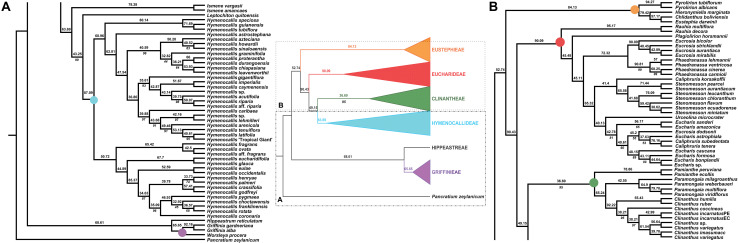
Local posterior probability (LPP) coalescent species tree from ASTRAL III analysis of gene trees of 526 nuclear genes. LPP scores appear above the branches; bootstrap percentages <100 shown below branches (all unmarked branches have 100% BP). The tree is divided into two sections **(A,B)** for readability; refer to the colored inset (a cartoon of the tree collapsed to the tribal level) as a guide. Colored circles at the nodes in the full tree conform to clade colors in the inset.

Within Hymenocallideae, *Ismene* was the first branch, followed by *Leptochiton*, and then *Hymenocallis*. The latter had a consistent topology of two subclades, both of which consisted of a small, pseudopetiolate-leafed, forest understory subclade sister to a larger, more diverse group (Figure 2). One pseudopetiolate group was sister to a clade containing all other West Indian and most of the Mesoamerican species in our sampling. Some of the more terminal subclades were either poorly or not supported (Figure 2). The West Indian *Hymenocallis caribaea* (L.) Herb. was the first branch with 100 BP (Figure 2) in the complete supermatrix and in the 90% coverage (Supplementary Figure 4), but formed an unsupported sister to *Hymenocallis gigantiflora* Meerow^3^ in the 70% coverage best tree (Supplementary Figure 3). The second pseudopetiolate subclade is sister to a clade that united two southern Mexican pseudopetiolate species, *Hymenocallis eucharidifolia* and *Hymenocallis glauca* M. Roem., with a monophyletic southeastern United States subclade (Figure 2). A small clade of mostly West Indian species resolved with 94 BP (Figures 2, 3), uniting *Hymenocallis arenicola*, *Hymenocallis latifolia*, *Hymenocallis tenuiflora* and a cultivar of Caribbean origin, *H*. ‘Tropical Giant.’

In Eucharideae, *Rauhia* was the first branch, followed by the monotypic Colombian endemic *Plagiolirion* (Figure 2). Next, *Eucrosia* (less *Eucrosia dodsonii* Meerow & Dehgan) and *Phaedranassa* formed sister clades (100% BP). *Stenomesson* was monophyletic and sister to *Caliphruria korsakoffii* (Traub) Meerow, the only Peruvian species of that genus. The remaining clade of Eucharideae united the Colombian *Caliphruria*, *Eucharis*, and *Urceolina*, with the inclusion of *E. dodsonii*. *Urceolina* was the first branch, though not consistently in all of the supermatrix analyses (Figure 2 and Supplementary Figures 2–4). *Eucharis* was not monophyletic. An unexpected and consistent subclade united *Eucharis astrophiala* (Ravenna) Ravenna, the Colombian *Caliphruria* spp., and *E. dodsonii* (97% BP).

A ML tree derived from a slightly different whole gene supermatrix (five samples missing) using ambiguity codes to account for all paralogs (Supplementary Figure 5) showed very strong congruence with trees from our paralog-pruned alignments (Figure 2 and Supplementary Figures 2–4).

### Species Tree Analyses

The ASTRAL-III species tree analyses (Figure 3) were largely congruent with the ML trees. *Pamianthe* was resolved in all species trees as the first branch within Clinantheae. The topology of all of the other major subclades was the same. LPP scores ranged from 33 to 94, but BP was high at the majority of nodes (Figure 3). Little change occurred with increased percentage coverage across taxa (Supplementary Figures 6, 7). There occurred some re-alignment within the two main subclades of *Hymenocallis*, mostly at nodes with low bootstrap support in the full data set (Figure 3) and some erosion of BP overall. The sister status of *Caliphruria korsakoffii* to *Stenomesson* was resolved but not supported by the 70% coverage species tree bootstrap (Supplementary Figure 6). In the 90% species tree, it was sister to the *Eucharis*/*Urceolina*/*Caliphruria*/*E. dodsonii* clade with weak (63%) BP (Supplementary Figure 7).

### Plastome Analysis

**FIGURE 4:**
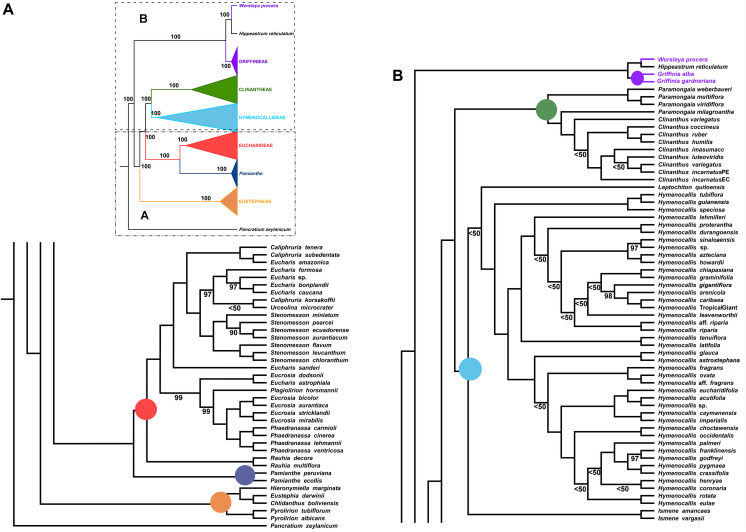
Best tree from maximum likelihood analysis of partial plastome sequences across the Andean tetraploid clade of Amaryllidaceae subfam. Amaryllidoideae, with bootstrap percentages <100 shown above branches (all unmarked branches have 100% BP). The tree is divided into two sections **(A,B)** for readability; refer to the colored inset (a cartoon of the tree collapsed to the tribal level) as a guide. Colored circles at the nodes in the full tree conform to clade colors in the inset.

The ML analysis of the partial plastome was congruent at the tribal clade level with the nuclear genome, but resolved yet a third resolution for *Pamianthe* as sister to Eucharideae with 100% BP (Figure 4). The relationships of the Eustephieae clade were identical to the nuclear trees (Figures 2, 3). *Paramongaia milagroantha* (S. Leiva & Meerow) Meerow was sister to *Clinanthus* (100%), and *C. incarnatus* did not resolve as monophyletic (Figure 4). Hymenocallideae had the greatest number of unsupported nodes. The southern Caribbean/Guianan clade of *Hymenocallis* (*Hymenocallis guianensis*, *Hymenocallis speciosa*, and *Hymenocallis tubiflora*) became the first branch in the genus (Figure 4) instead of sister to the Caribbean/Mexican subclade as with nuclear data (Figures 2, 3). In the other subclade of *Hymenocallis*, *Hymenocallis astrostephana* T. M. Howard/*H. glauca* became the first branch (Figure 4) with no support, followed by the West Indian pseudopetiolate clade. A SE U.S. clade received 100% support (Figure 4), but had poor internal resolution. It was sister to a group of mostly Mexican spp. that resolved within the other main subclade with nuclear genes (Figures 2, 3). *Hymenocallis caribaea* was a well-supported sister sp. to *H*. ‘Tropical Giant’ (100% BP), which joined with *H. arenicola* and *H. gigantiflora* in a well-supported subclade (98% BP). *H. latifolia* and *H. tenuiflora* (100% BP) were also the first branch in a clade of mixed Mexican and West Indian taxa.

In the Eucharideae, a monophyletic *Stenomesson* was embedded within *Eucharis*, *Caliphruria*, and *Urceolina* (Figure 4), and *E. sanderi* Baker was the first branch. *C. korsakoffii* was sister to *Urceolina microcrater* Kraenzl. without support. *E. astrophiala* was sister to *E. dodsonii*, together in turn resolved as sister to a *Plagiolirion*/*Eucrosia*/*Phaedranassa* clade. *Rauhia* was the first branch in the tribe after *Pamianthe*. We also analyzed only the coding and tRNA regions of the plastome and obtained the same topology as Figure 4 with variably increased or decreased BP at various nodes (not shown).

### Cytonuclear Discordance

**FIGURE 5:**
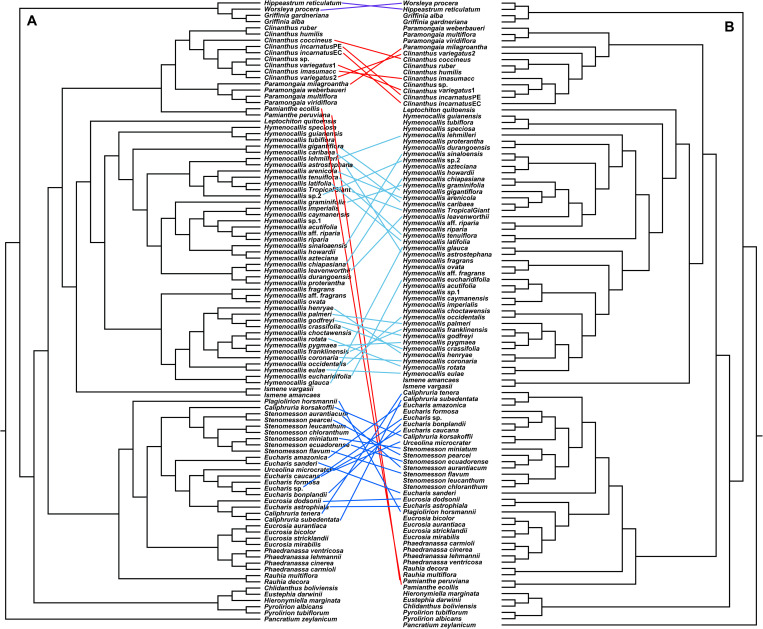
“Tanglegram” created from the best ML 70% taxon coverage nuclear supermatrix (Supplementary Figure 3) and the plastome trees (Figure 4) graphically showing cytonuclear discordance between the two partitions. A = nuclear tree, B = plastome tree.

The congruence between supermatrix and species tree topologies of our nuclear genes (Figures 2, 3 and Supplementary Figures 2–4, 6, 7) suggested that there was no reticulation between major lineages (tribes) of the Andean tetraploid clade, with the possible exception of *Pamianthe* (see section “Discussion”). Our plastome data (Figure 4) resolved the same very well supported tribal clades as the nuclear data. However, the tanglegram (Figure 5) clearly illustrated cytonuclear discordance within the genera *Clinanthus* and *Hymenocallis*, and in the tribe Eucharideae, particularly among the genera *Caliphruria*, *Eucharis*, and *Urceolina*. None was observed for sister genera *Eucrosia* and *Phaedranassa*. There was also a disagreement on the position of *Worsleya procera* relative to the other species from the hippeastroid clade (Figure 5). The plastome tree resolved this monotypic genus as sister to *Hippeastrum reticulatum*, while the nuclear trees placed it within the *Griffinieae*.

### Species Network Analysis

**FIGURE 6:**
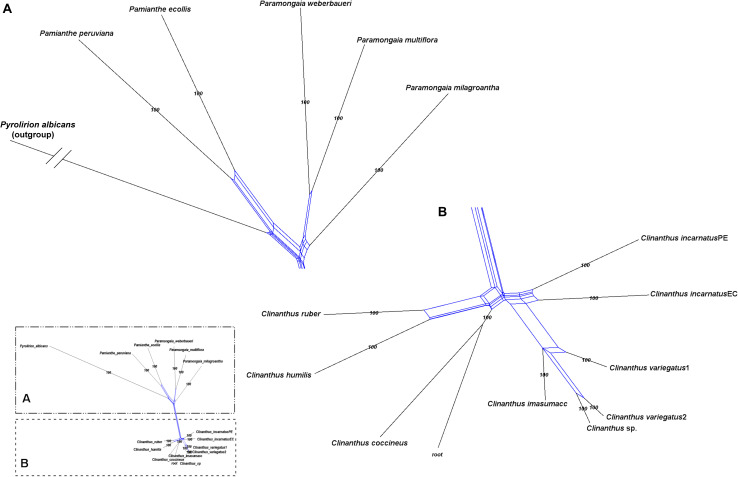
Equal angle 360° hybridization network generated by SplitsTree v. 4.15.1 based on the 99% taxon coverage supermatrix (70 nuclear genes) across the tribe Clinantheae. Numbers above branches are percentages from 300 bootstrap iterations. The entire network is shown in the inset, with subclades **(A)** and **(B)** magnified for clarity.

Hybridization networks of Clinantheae, Eucharideae, and *Hymenocallis* supported varying degrees of putative reticulation within each clade, and the phi test detected significant recombinant signal in all three supermatrices at *p* = 0.0. All branches in the networks were strongly supported by the bootstrap. Clinantheae (Figure 6) had the least amount of putative hybridization, and none between genera. There was some minor reticulation evident between *Pamianthe ecollis* Silverst., Meerow & Sánchez-Taborda, and *P. peruviana* and between *Paramongaia multiflora* Meerow and *Paramongaia weberbaueri* Velarde. Recombinant signal was highest among *C. variegatus, Clinanthus imasumacc* (Vargas) Meerow and related species, but not between them and *C. incarnatus*, the latter exhibiting genetic exchange between Peruvian and Ecuadorian collections. Minor reticulation was also apparent between *Clinanthus humilis* (Herb.) Meerow and *Clinanthus ruber* (Herb.) Meerow & A. Cano.

**FIGURE 7:**
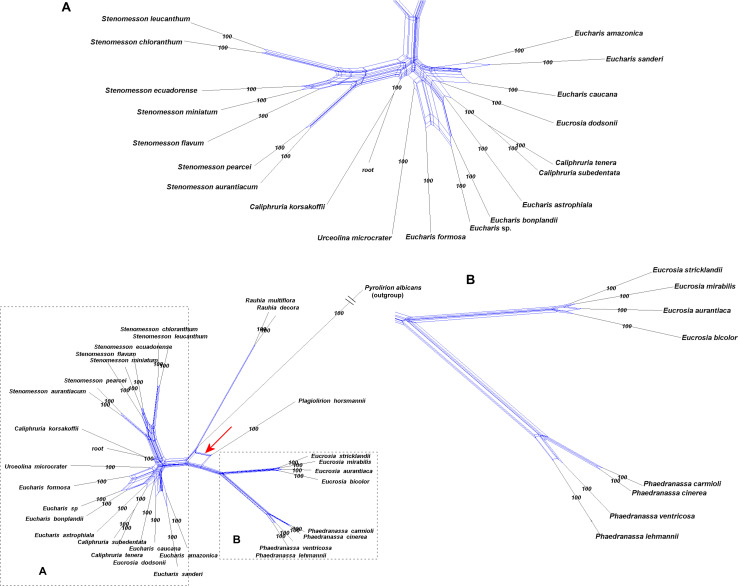
Equal angle 360° hybridization network generated by SplitsTree v. 4.15.1 based on the 99% taxon coverage supermatrix (70 nuclear genes) across the tribe Eucharideae, with percentages from 300 bootstrap iterations. The entire network is shown in the inset, with subclades **(A)** and **(B)** magnified for clarity. Red arrow indicates possible reticulate origin of the monotypic *Plagiolirion*.

Within the Eucharideae, there was only minor reticulation between sister species of *Eucrosia* and *Phaedranassa* (Figure 7), respectively, with considerably more evident among species of *Stenomesson*, and possibly in the early diversification of *Stenomesson* from the *Caliphruria*/*Eucharis*/*Urceolina* subclade (i.e., position of *C. korsakoffii*). Recombinant signal suggested that the monotypic *Plagiolirion horsmanii* Baker may have arisen via reticulation between the branch leading to *Eucrosia* and *Phaedranassa* and the ancestral lineage of *Rauhia*. However, the reticulate origins of *Eucharis*, Colombian *Caliphruria*, *Urceolina*, and *E. dodsonii* were strongly evident, as was the misdiagnosis of the latter as a species of *Eucrosia*.

**FIGURE 8:**
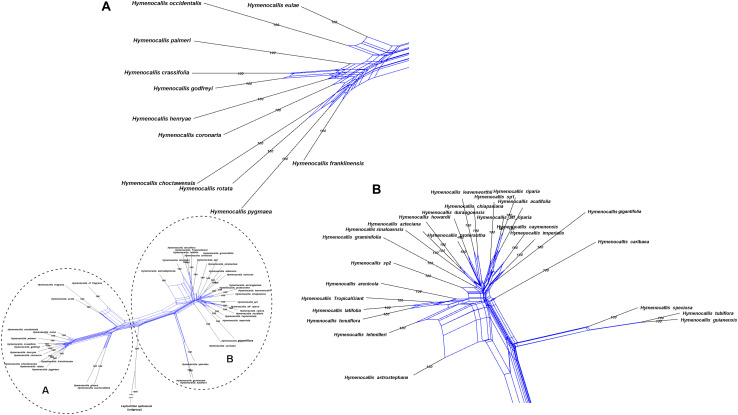
Equal angle 360° hybridization network generated by SplitsTree v. 4.15.1 based on the 99% taxon coverage supermatrix (70 nuclear genes) across the genus *Hymenocallis*, with percentages from 300 bootstrap iterations. The entire network is shown in the inset, with subclades **(A)** and **(B)** magnified for clarity.

*Hymenocallis* exhibited the greatest degree of interspecific reticulation in our network analyses (Figure 8). Some minor putative recombination signal occurred in both of the small pseudopetiolate subclades. But within both the larger SE U.S. subclade and the Mesoamerican/West Indian subclade, strong interspecific reticulate patterns were evident.

### Dating and Diversification Rate Analyses

**FIGURE 9:**
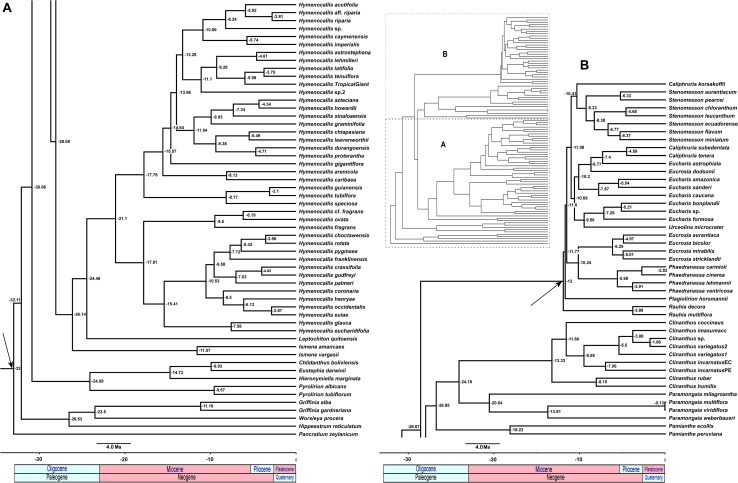
Relaxed penalized likelihood chronogram of the Andean tetraploid clade using the chronos function of APE with the best 90% taxon coverage supermatrix tree found by RAxML. Arrows indicate calibration nodes used in the analysis. Numbers along branches are millions of years before present. The entire tree is shown in the inset, with subclades **(A)** and **(B)** magnified for clarity.

**FIGURE 10:**
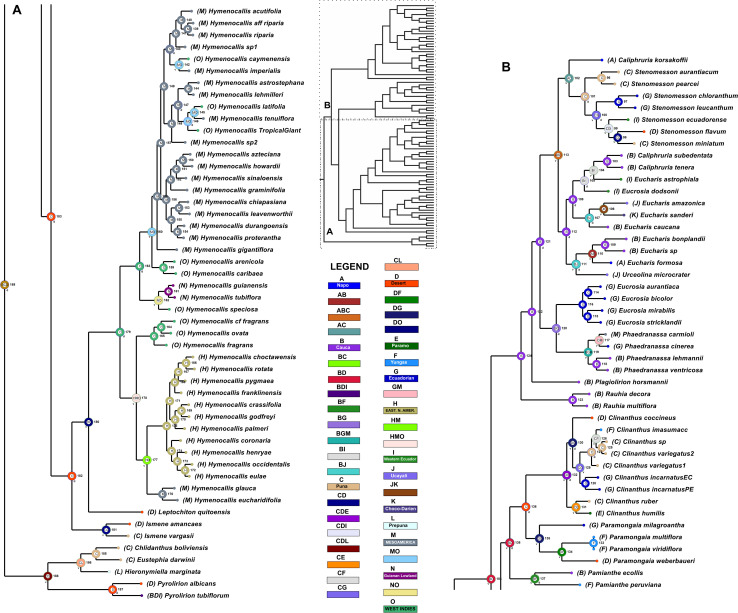
Area cladogram generated by Divalike+J analysis with BioGeoBears through RASP 4.2, using the best tree found by maximum likelihood of the 90% taxon coverage supermatrix with RAxML. Areas at any node were limited to three, and only the most likely was mapped onto the tree. Lower case “v” and “d” adjacent to a node = vicariance or dispersal, respectively. The tree is divided into two sections **(A,B)** for readability; refer to the inset as a guide.

The relaxed model was much less sensitive to the value of λ than the correlated, and several trees had equally high penalized likelihood scores. We therefore used the tree calibrated under the relaxed model and λ = 1 for further analyses (Supplementary Figure 8). The resulting tree (Figure 9), dated the crown node of the Andean clade at 30.9 Mya (stem = 32.1 Mya), thus an early Oligocene origin, with the first branch (Eustephieae) crowning at around 24 Mya in the late Oligocene (Table 1). The MRCA of Clinantheae and Hymenocallideae was dated at 28 Mya, and both tribes’ crown ages are ca. 26 Mya. *Ismene* and *Leptochiton* diverged from *Hymenocallis* by the late Oligocene (ca. 26 and 24.5 Mya, respectively). The crown age of *Hymenocallis* was ca. 21 Mya, and the two main subclades were contemporaneous at almost 18 Mya. *Pamianthe* diverged from *Clinanthus* and *Paramongaia* ca. 27 Mya, with a Miocene crown age of ca. 18 Mya. *Paramongaia* and *Clinanthus* split in the late Oligocene (Figure 10), but the former is older than *Clinanthus* by ca. 8 MY. The very recent divergence between *P. multiflora* and *P. viridiflora* (ca. 130 Ky) may be somewhat artefactual as only eight genes were successfully captured from the latter, which was extracted from an herbarium sample. While the stem node of Eucharideae appeared as late mid-Oligocene (ca. 28 Mya), its generic radiation was a Miocene phenomenon, occurring rapidly between the mid-Miocene through the Pliocene (Figure 9 and Table 1). There was very sudden radiation of lineages between 10 and 11 Mya (Figure 9).

**TABLE 1:** Estimated ages of major clades of the Andean tetraploid clade using penalized likelihood with the R package ape on the on the best 90% taxon coverage supermatrix tree found by maximum likelihood with RAxML.

**Clade**	**Stem age**	**Crown age**
Clinantheae	28.08	26.85
*Clinanthus*	24.18	13.33
*Pamianthe*	26.85	18.22
*Paramongaia*	24.18	20.64
Eucharideae	28.67	12 (calibration node)
*Caliphruria*/*Eucharis*/*Urceolina*	11.08	10.69
*Eucrosia*	10.24	6.25
*Phaedranassa*	10.24	5.68
*Plagiolirion*	11.77	–
*Rauhia*	12 (calibration node)	3.88
*Stenomesson* (inc. *Caliphruria korsakoffii*)	11.08	10.41
Eustephieae	30.86	24.09
*Chlidanthus*	9.93	–
*Eustephia*	9.93	–
*Hieronymiella*	14.73	–
*Pyrolirion*	24.09	9.57
Hymenocallideae	28.06	26.14
*Hymenocallis*	24.46	21.1
*Ismene*	26.14	11.57
*Leptochiton*	24.46	–

*Ages are My before present (Figure 9).*

Both ESS scores of our BAMM run were >3000, indicating good convergence across the MCMC generations and number of rate shifts sampled. Post run analyses with BAMMtools all supported the null model of no rate shifts, thus the absence of rate-shift heterogeneity in the Andean tetraploid clade.

### Biogeographic Analysis

The DIVAlike model had the highest weighted AICc score of the six models tested with BioGeoBears, and probability values supported the +J add-on (founder effect speciation); thus that model was applied to our tree (Figure 10). Globally, the model supported 36 dispersal events, 32 vicariance events and no extinction (Supplementary Table 3). The largest number of dispersals originated from the Desert Province (10.17), followed by Cauca Province (9.17). Puna Province was the destination for the largest number of dispersals (4.0) The Mesoamerica Bioregion had the largest number of speciation events within an area (18), followed by the Eastern North America bioregion (10), no doubt due to the relatively large number of samples of *Hymenocallis* endemic to both areas. Cauca Province was third, with nine.

The ancestral area (node 189, *p* = 0.0565, Figure 10) encompassed the Cauca, Desert and Puna Provinces (BCD). Two events took place from this node; dispersal into the Prepuna Province (L) at the crown node of Eustephieae (188, *p* = 0.3340), from which representation within the Cauca Province was eliminated, and vicariance between the Desert (D) and Puna (C)/Prepuna (L) Provinces. At node 187 (the crown node of *Pyrolirion*, *p* = 0.7468) two dispersals take place into Cauca and Western Ecuador Provinces. Vicariance between the Puna (C) and Prepuna (L) Provinces occurs at node 186 (*p* = 0.9847). At node 184 (*p* = 0.2534), vicariance between the Cauca (B) and Desert (D) Provinces separates the stem node of Hymenocallideae and Clinantheae (183) and the crown node of Eucharideae (124), respectively. From node 183 (*p* = 0.1040), a return dispersal to Cauca Province took place to the crown node of Clinantheae (138, *p* = 0.9030), while the crown node of Hymenocallideae (182, *p* = 0.7938) remains optimized in the Desert Province (D), followed by a range expansion to Puna Province (C) at the crown node of *Ismene* (181, p = 0.9696), after which vicariance separates these two provinces between the two species of *Ismene* in our sampling. From this same node, a second, presumably long distance, dispersal to the West Indies Bioregion (O) occurs at the stem node of *Hymenocallis* (180, *p* = 0.8065), with subsequent vicariance between the West Indies (O) at the crown node of *Hymenocallis* (179, *p* = 0.2944) and the divergence of *L. quitoensis*. From the crown of *Hymenocallis*, dispersal to the Eastern North America (H) and Mesoamerican (M) Bioregions occurs (178, *p* = 0.3234), with subsequent vicariance between the West Indies (165, *p* = 1.0000), and both the Mesoamerica and Eastern North American Bioregions (177, *p* = 0.9455) in one of the two subclades of *Hymenocallis*, followed by vicariance between the two latter bioregions (nodes 175 and 176, *p* = 1.0000 for both). The lack of either vicariance or dispersal at nodes 166–175 suggests that the entry of the genus into the SE U.S. was a solitary event.

From the West Indian crown node of the second subclade of *Hymenocallis* (163, *p* = 0.8308), two dispersals expanded the range to the Guianan Lowlands Province (162, *p* = 0.9499) and the Mesoamerican Bioregion (160, *p* = 0.9277), followed by vicariance between the two areas. The remaining events in *Hymenocallis* were (1) secondary dispersals to the West Indies Bioregion from Mesoamerica (143, 147; *p* = 0.9030, 0.3412), followed by vicariance (142, 145; p = 0.9052, 0.8709.

From the crown node of Clinantheae (138, *p* = 0.1060) in the Cauca (B) and Desert Provinces (D), dispersal to the Yungas Province took place at the crown node of *Pamianthe* (137, *p* = 0.9642), while vicariance restricted the range of the stem node of *Clinanthus* and *Paramongaia* (136, *p* = 0.0621) to the Desert Province (D). Vicariance subsequently isolated *P. ecollis* in Cauca Province and *P. peruviana* in Yungas Province. The crown node of *Paramongaia* (135, *p* = 0.4363) resolved within the Desert (D) and Ecuadorian (G) Provinces, from where it dispersed into Yungas Province (134, *p* = 0.9680) while vicariance separated *P. milagroantha* from the other spp. The crown node of *Clinanthus* (132, *p* = 0.0956) was optimized within the Puna (C), Desert (D) and Páramo (E) Provinces. The genus dispersed to the Ecuadorian Province and vicariance separated the crown nodes of the two subclades into a Desert and Ecuadorian group (DG, 130, *p* = 0.3268) and a Puna and Páramo (CE) group (131, *p* = 0.9250). Vicariance split the latter small subclade of *C. ruber* and *C. humilis* between the two provinces. There are a number of additional species with affinity to this group that were not sampled. The larger clade of *Clinanthus* extended its range into the Ecuadorian (G) Province (130, *p* = 0.3268), and *C. coccineus* was isolated by vicariance in the Desert Province. The group then dispersed into Puna Province (129, *p* = 0.8733), followed by vicariance between the Puna and Ecuadorian Provinces (127, *p* = 0.8077; 128, *p* = 1.0000), and a terminal range extension by *C. imasumacc* into Yungas Province.

The crown node of Eucharideae (124, *p* = 0.9666) was assigned to Cauca Province, and no subsequent events occurred until node 121 (*p* = 0.1035) when three dispersals extended the range at node 120 (*p* = 0.2882) to the Ecuadorian Province and to Napo (A) and Puna (C) Provinces at node 113 (*p* = 0.2329). At node 120, range extension into Mesoamerica occurs at the crown node of *Phaedranassa* (119, *p* = 0.5591), with subsequent vicariance between Cauca Province (118, *p* = 1.0000), and the Ecuadorian Province/Mesoamerica Bioregion (117, *p* = 0.9663). It should be noted that the majority of *Phaedranassa* species are endemic to the Ecuadorian Province, of which only one is represented here. The *Eucrosia* clade remains within the Ecuadorian Province (114–116, *p* = 1.0000). Vicariance occurs at node 113 (*p* = 0.2329) between Cauca (112, *p* = 0.3327) and Napo/Puna Provinces (102, *p* = 0.6748). From node 112, the crown node of the “Eucharis-Urceolina” clade, dispersal takes place to Ucayali Province (111, *p* = 0.6193) with a mosaic of subsequent dispersals and vicariant events taking place between that province, Western Ecuador (I), Cauca (B), Napo (A), and Chocó-Darién (K) Provinces (nodes 103-111, see Supplementary Table 3 for probabilities) among the species of *Eucharis*, *Caliphruria*, and *Urceolina* (and *E. dodsonii*) represented in this subclade. The second subclade (102), the crown node of *Stenomesson* (including *Caliphruria korsakoffii*), was marked by dispersal and vicariance events involving Desert (*Stenomesson flavum* Herb., Ecuadorian [*Stenomesson chloranthum* Meerow & van der Werff, *Stenomesson leucanthum* (Ravenna) Meerow & van der Werff], Puna (*Stenomesson aurantiacum* Herb., *Stenomesson miniatum*, *Stenomesson pearcei* Baker), and Western Ecuador (*Stenomesson ecuadorense*) Provinces; see Figure 10 and Supplementary Table 3 for details.

For the plastome tree, the BAYAREAlike model had the best weighted AICc score with BioGeoBears, and probability value supported the +J option, thus that model was applied to the tree (Supplementary Figure 9). The number of dispersal events = 67, vicariance = 33, and there was a single extinction. This extinction event occurred at node 107, the stem node of *Stenomesson*, with the elimination of putative species in the Cauca Province at the crown node of the genus. Probabilities for each event were considerably higher for the plastome tree (Supplementary Table 4) compared to the nuclear supermatrix tree (Supplementary Table 3), with much fewer compound distributions at ancestral nodes. Most notably, Western Ecuador Province, rather than Puna Province was designated as one of three ancestral areas at node 189 in the plastome tree. The crown node of *Hymenocallis* is situated with greatest likelihood within the Mesoamerica Bioregion (Supplementary Figure 9), though both the Guianan Lowland Province and West Indies Bioregion are also included (with lower likelihood) if all potential areas are shown. See Supplementary Table 4 for details.

## Discussion

### Overview

Both nuclear and plastome genomics leave no doubt that the recognition of four monophyletic tribes, Clinantheae, Eucharideae, Eustephieae, and Hymenocallideae, within the Andean tetraploid clade of Amaryllidaceae subfam. Amaryllidoideae is a highly supported, and stable evolutionary classification for the group (Meerow et al., 2000; Meerow, 2010; Meerow and Nakamura, 2019). Only the position of *Pamianthe* retains ambiguity (Figures 2–4), which in the nuclear phylogenomic data is eliminated by the coalescent species tree (Figure 3) and supermatrices with less missing data (Supplementary Figures 3, 4). Plastome data support a third possible resolution for this enigmatic genus of three epiphytic or lithophytic species (Meerow et al., 2019), as sister to Eucharideae (Figure 4). This is discussed below under “Clinantheae.” Chase et al. (2009) recognized both tribes Eucharideae and Stenomesseae (which we consider synonymous), but not Clinantheae. As they did not list component genera for the tribes of subfam. Amaryllidoideae, it is impossible to determine how they circumscribed these tribes. While there is only minor incongruence between the supermatrix (Figure 2 and Supplementary Figures 2–4) and the coalescent species trees (Figure 3 and Supplementary Figures 6, 7), strong cytonuclear incongruence is evident at the intra-tribal and generic levels (Figure 5).

Our diversification analysis robustly found no rate shifts anywhere in the Andean tetraploid clade, and the nuclear gene biogeographic analysis did not detect any extinction events. A high number of extinction events can be a trigger for diversification rate shifts (Brocklehurst et al., 2015), and in that sense, these two analyses mutually support the relative absence of both in the history of the Andean Amaryllidaceae, but may indicate that an Oligocene origin is an over-estimation of the age of the clade.

### Cytonuclear Discordance

Cytonuclear discordance is the phenomenon wherein phylogenies inferred from nuclear genes differ markedly from trees constructed using organellar genes (Rieseberg and Soltis, 1991; Folk et al., 2017; Morales-Briones et al., 2018; Lee-Yaw et al., 2019). Whole or partial plastomes have increasingly been used for phylogenetic analysis, and when contrasted with various large nuclear gene sets, cytonuclear discordance is frequently encountered (Huang et al., 2014; Bruun-Lund et al., 2017; Folk et al., 2017; Sloan et al., 2017; Morales-Briones et al., 2018). Cytonuclear discordance may arise via (1) incomplete lineage sorting (ILS) of ancestral polymorphisms, such that phylogenetic relationships from organellar markers do not capture a true evolutionary history of the taxa under study (Maddison and Knowles, 2006; Joly et al., 2009; Stolzer et al., 2012; Solís-Lemus and Ané, 2016; Knowles et al., 2018), (2) selection operating within organellar genomes independent of speciation (Sloan et al., 2017; Lee-Yaw et al., 2019), and (3) hybridization and/or chloroplast transfer, upon which selection may also play a role (García et al., 2017; Sloan et al., 2017; Bastide et al., 2018; Degnan, 2018; Folk et al., 2018; Morales-Briones et al., 2018). Hippeastreae subtribe Hippeastrinae exhibited a great deal of cytonuclear discordance, but also showed combined effects of ancient reticulation and incomplete linkage sorting (García et al., 2014, 2017). While there was initial evidence from nrDNA ITS sequences of early reticulation in Hippeastreae (Meerow et al., 2000; Meerow, 2010; García et al., 2014, 2017), there was none in the Andean tetraploid clade (Meerow, 2010), although the clade had lower sampling taxonomically and genetically than our current study.

The hybridization network (Figure 7) suggests ancestral reticulation within the subclade of Eucharideae (Figure 7) containing *Eucharis*, *Caliphruria* (except *C. korsakoffii*), *Urceolina*, and the surprising inclusion of *E. dodsonii*, which has consequences for the taxonomy of the subclade (discussed below under “Eucharideae”). The only other putative reticulate origin at the generic level is the monotypic *Plagiolirion* (Figure 7). We are inclined to weight the agreement of supermatrix and coalescent analytical approaches to our nuclear data as evidence that ILS has been rare in the evolution of the Andean tetraploid clade as has been argued with other plant taxa (Stephens et al., 2015; Folk et al., 2017) as well as theoretically (Bayzid and Warnow, 2013; Roch and Warnow, 2015). The two exceptions might be the genus *Pamianthe*, which resolved in three different places among our three main trees (Figures 2–4), and perhaps the apparent reticulate origin of the Colombian endemic monotypic *Plagiolirion* (Figure 8). However, the supermatrices with less missing data (Supplementary Figures 2, 3) are in agreement with the species tree (Figure 3) that *Pamianthe* is the first branch of the tribe Clinantheae. We thus favor reticulation over ILS as the primary cause of observed cytonuclear discordance (Figure 5), with *Pamianthe* the most likely instance of ILS, and perhaps *Plagiolirion* as well.

The resolution of *Worsleya* as sister to *H. reticulatum* in our plastome tree could be evidence that *Worsleya* represents a hybrid of *Griffinia* and *Hippeastrum*, and was previously observed but unreported (N. García, pers.comm.). Though outside of the scope of this paper, this discordance needs to be further analyzed with greater sampling of Hippeastreae and Griffinieae.

We now discuss each of the four resolved monophyletic tribes of the Andean tetraploid clade, placing our phylogenetic and biogeographic inferences into a clade-based context.

### Eustephieae

The first branch of the Andean tetraploid clade constitutes the tribe Eustephieae, which consists of four genera, *Chlidanthus*, *Eustephia*, *Hieronymiella*, and *Pyrolirion*. The staminal corona is absent (*Pyrolirion*), reduced to basal connation of the subulate filaments (*Chlidanthus*), present, but modified (*Hieronymiella*) or reduced to basal connation, but with acute appendages distally on both sides of the primarily filiform filaments (*Eustephia*). The uniflorus *Pyrolirion* has the broadest distribution of the four, occurring from Ecuador to Bolivia, on the western slopes and inter-Andean valleys, predominantly within the Prepuna and Puna Provinces, but also the Peruvian coastal fog desert *lomas* (Desert Province). The style is branched apically in this genus. Due to uniflory, fused spathe bracts, and perigone shape, it was believed to be allied with *Zephyranthes* Herb. until recently (Meerow, 2010). *Pyroliron albicans* bears superficial resemblance in floral morphology to the uniflorus *Hippeastrum* Herb. subg. *Tocantinia* (Ravenna) Nic. García (García et al., 2019). Huaylla et al. (in press) lists eight species for the genus, but this may not reflect synonym in the absence of a thorough revision (Cowley, 1989).

The Eustephieae represents the southernmost element of the Andean tetraploid clade, outside of the reported occurrence of *C. humilis* in the Andes of northern Chile (Ravenna, 1974a). It is the least well understood of the Andean tetraploid clade tribes, and lacks one of three indel regions of nrDNA ITS that are apomorphic for the rest of the Andean clade (Meerow et al., 2000).

*Eustephia*, with perhaps four species, is restricted to southern Andean Peru (Puna Bioprovince). The majority of *Hieronymiella* spp. are found in the seasonally arid Prepuna Province (Cabrera, 1994) of northern Argentina (Arroyo-Leuenberger and Leuenberger, 2004), with extensions into montane central and southern Bolivia (Lara and Huaylla, 2015; Lara, 2018). There are six to eight species in the genus (Arroyo-Leuenberger and Leuenberger, 2004; Lara and Huaylla, 2015; Lara, 2018), which show triploid and aneuploid derivations of the ancestral 2*n* = 46 chromosomes (Di Fulvio, 1973). It is most diverse genus of the tribe in terms of floral morphology (Arroyo-Leuenberger and Leuenberger, 2004). *Chlidanthus* is found only in the *lomas* of Central and southern Peru (Desert Province) and Andean Bolivia (Lara and Céspedes, 2019, Puna Province). All of the 3–4 species have long-tubed yellow flowers.

In our reconstruction, a gap of ca. 6 Mya separates the stem and crown nodes of Eustephieae, suggesting a period of stasis until the other three genera diverged from *Pyrolirion* (Figure 9). The tribe may have originated in the Desert, Prepuna, or Puna Provinces comprising Central and/or southern Peru and perhaps Bolivia (Figure 10). The accuracy of biogeographic scenarios in Eustephieae is compromised by limited sampling of all of the genera with presence in more than one biogeographic area. Nonetheless, it is likely that diversification of the tribe was promoted by the uplift of the eastern cordillera of the central Andes starting in the Miocene (Garzione et al., 2008; Antonelli et al., 2009; Luebert and Weigend, 2014), which might account for the predominance of vicariant events in the internal nodes of the Eustephieae phylogeny (Figure 10). That uplift in Andean Peru may have proceeded from the south to the north (Picard et al., 2008) probably influenced the diversification timing of the tribe relative to the other groups in the Andean clade.

### Clinantheae

According to our results, the Clinantheae may have diverged from its sister clade Hymenocallideae in the Oligocene (Figure 9), with a crown origin in the Cauca and Desert Bioprovinces (Figure 10). The first branch, *Pamianthe*, is difficult to parse biogeographically because the exact locality of *P. peruviana* in Peru is unknown, and we have no sequence data for the Ecuadorian endemic *P. parviflora* Meerow (Meerow, 1984). Meerow and Nakamura (2019) and Meerow et al. (2019)proposed that the genus, with its broad range from Colombia to Bolivia may indeed represent a relictual epiphytic and lithophytic lineage that colonized cloud forests of the western Andes that have been in retreat since at least the Miocene and have also suffered large-scale and largely undocumented destruction (Dillon et al., 1995; Mutke et al., 2017). Both *Clinanthus* and its sister genus *Paramongaia* originated in the Desert Province, and diversified in the Ecuadorian, Paramo, Puna, and Yungas Provinces, largely within Peru, both dispersing into Bolivia, but only *Clinanthus* into southern Peru and Ecuador, although not until the Pliocene (Figure 9). *Clinanthus* is most diverse in northern and central Peru. The northern Peruvian (Ecuadorian Province) spp. of *Paramongaia* [*P. milagroantha* and *P. mirabilis* (Ravenna) Meerow], the latter not available for the sequence capture, but sister to *P. milagroantha* with ITS (Meerow and Nakamura, 2019), were isolated from the rest of the genus in the early Miocene.

*Clinanthus* separated into two sister subclades discussed by Esquerre and Meerow (2020) that diverged about 13 Mya in our analysis. The larger of the two conforms to *Stenomesson* subg. *Fulgituba* Ravenna as conceived by Ravenna (1974b) in his polyphyletic concept of that genus. The species of this subclade typically have lorate leaves, generally >1 cm. wide, long funnelform-tubular flowers, the tubes long relative to the limb, and various patterns of green in the relatively short limb. They occur in a diversity of habitats (Esquerre and Meerow, 2020). The second subclade typically has narrowly lorate to linear leaves <1 cm wide, and usually no green on the tepals. They appear younger than then the first group (Figure 9), and include the alpine species *C. humilis*, which occurs at over 4,000 m elevation and maintains its scape inside the bulb until seed capsule maturity (Meerow, 1987b). The genus as a whole may number two dozen or more species (León et al., 2013; Meerow and Cano, 2019; Esquerre and Meerow, 2020), many of which are known from single localities.

### Hymenocallideae

The Hymenocallideae is the one lineage of the Andean tetraploid clade that has extended its range the furthest into the West Indies, Mesoamerica and Eastern North America Bioregions. The complex taxonomic history of the tribe is reviewed by Meerow et al. (2002). It is a tenable although untested hypothesis whether long distance dispersal was facilitated by the curious fleshy seeds with photosynthetic integuments and starch storing embryos adapted for hydrochory (Whitehead and Brown, 1940; Flint and Moreland, 1943), relative to the flattened, winged, and presumably wind dispersed seeds of Clinantheae, most Eucharideae, and Eustephieae.

In our reconstruction, the tribe diverged from Clinantheae in the Oligocene, about 28 Mya (Figure 9), perhaps in the Desert Province of northern Peru, dispersing to Puna Province first, ca. 2 My later, the stem age of *Ismene*, which is the first branch of the tribe. Incomplete sampling might bias the area optimization of *Ismene*, other species of which range to Ecuador to the north, and southern Peru and Bolivia to the south (Meerow and Cano, 2019), but they remain primarily in the higher elevation Puna Bioprovince. *Leptochiton* diverged 2 My after *Ismene*. The genus is endemic to the Desert Province, specifically the low elevation Tumbes-Piura dry forest of NE Peru and SE Ecuador (Leal-Pinedo and Linares-Palomino, 2005; Linares-Palomino, 2006; Loaiza, 2013), with a very short season of active growth. Snoad (1952) reported 2*n* = 24 chromosomes for *L. quitoensis*; we counted 2*n* = 34 (unpubl. data). These would be the lowest reported somatic chromosome numbers in the entire Andean clade, and may actually represent karyotype reduction, which might have had some adaptive value to the Tumbes-Piura biome, considered the most xeric of the seasonally dry tropical forest types in South America (Loaiza, 2013). Meerow et al. (2002) considered *Leptochiton* the least derived genus of the Hymenocallideae because of its numerous ovules per locule and the presence of phytomelanin in the seed testa, but neither nuclear phylogenomics nor plastomics support this (Figures 2–4).

The ancestor of *Hymenocallis* managed to disperse to the West Indies Bioregion sometime between 21–24 Mya (Figures 8, 9), certainly the greatest instance of long distance dispersal in the Andean clade. Each of the two small, pseudopetiolate clades that resolve as first branches in their respective clades of *Hymenocallis* (Figures 2, 3) were established by the start of the upper Miocene (Figure 9). There is no evidence from the hybridization network (Figure 8) that there was reticulation between the two large subclades of the genus, though our partial plastome tree (Figure 4) resolves the *H*. *guianensis*/*speciosa*/*tubiflora* clade as the first branch in the genus, instead of sister to the subclade that contains the Eastern North American clade, and brings additional Mesoamerican species into this subclade.

The nuclear tree suggests that *Hymenocallis* entered Mesoamerica twice from the West Indies, establishing a small beachhead in the Mexican southern states of two species retaining pseudopetiolate leaves, *H. eucharidifolia* Baker in wet forests and *H. glauca* in drier habitats, before dispersing, presumably just once, to Eastern North America (Figure 10), either a second instance of long distance dispersal, or a gap in our sampling. All of the other Mesoamerican species that we sampled resolved in the second subclade (Figures 3, 4), which dispersed from the West Indies Province (Figure 10). There were either returns to the West Indies in the later history of this clade, or the mosaic was the consequence of reticulation (Figure 8). The alternative position for the *H. guianensis*/*speciosa*/*tubiflora* subclade in the BioGeoBears analysis of the best plastome ML tree (Supplementary Figure 9) places the crown node of the genus in the Mesoamerica Province, (Supplementary Figure 9), the product of a single dispersal. This may be a more realistic scenario of how *Hymenocallis* made its entry into Mesoamerica, given the degree of reticulation revealed by nuclear genes (Figure 8).

With the exception of Bush et al. (2010), previous molecular phylogenetic studies of *Hymenocallis* included no more than five species (Meerow et al., 2000, 2002). Bush et al. (2010) utilized a combination of 23 ISSR presence/absence and 10 qualitative and quantitative morphological characters to examine relationships among the 15 endemic species of the genus from the Eastern North American Bioregion recognized by Smith (Smith and Garland, 2003); one additional was recently described (Smith and Garland, 2009). Our data (Figures 2, 3) agreed with some of the conclusions reached by Bush et al. (2010) with combined ISSR and morphological data, e.g., the sister relationship of *H. henryae* Traub and *Hymenocallis palmeri* S. Watson, the inclusion of *Hymenocallis eulae* Shinners as a synonym of *Hymenocallis occidentalis* (Leconte) Kunth (Smith and Garland, 2003), placement of *Hymenocallis choctawensis* Traub, *Hymenocallis franklinensis* G.Lom.Sm., L.C.Anderson & Flory, and *Hymenocallis pygmaea* Traub in the same subclade, but was completely at odds with their resolution of *Hymenocallis crassifolia* Herb., *Hymenocallis godfreyi* G.Lom.Sm. & Darst, and *Hymenocallis rotata* (Ker Gawl.) Herb. While the Bush et al. (2010) tree was congruent with the Traub (1962) system of “alliances” (Caroliniana and Henryae) for the endemic Eastern North American taxa, our cladistic resolution of the clade was not as straight forward. Traub (1962, 1980) recognized four additional alliances for the rest of the genus. Our data indicate that all four are para- or polyphyletic.

In addition to the unusual seed morphology, the seeds of *Hymenocallis* can be polyembryonic (Traub, 1966; Borys et al., 2005). The genus also exhibits high instances of polyploidy and aneuploidy (Flory and Schmidhauser, 1957; Flory, 1976) from the ancestral 2*n* = 46 (Flory, 1976), which may be further evidence of interspecific hybridization. These biological characteristics of the genus when coupled with the ability to reproduce vegetatively, may confound a complete understanding of the systematics of the genus.

### Eucharideae

This monophyletic tribe represents the pseudopetiolate genera of the Andean tetraploid clade. This character, which is broadly homoplasious throughout the family (Meerow and Snijman, 1998), and evolved at least twice in *Hymenocallis*, became fixed in this group, along with the loss of *ndh*F from the plastome (Meerow, 2010), and pseudogenization of other members of the *ndh* family (Supplementary Table 5). The short branch lengths of the stem lineages suggest a rapid mid-Miocene radiation (Figure 9), perhaps a consequence of the Andean orogeny (Gentry, 1982) and/or environmental heterogeneity and spatial dynamics (Mutke et al., 2014).

From an ancestral area in the Cauca Bioprovince (Figure 10), *Rauhia* diverged from the rest of the genera in the mid-Miocene (Figure 9). *Rauhia*, found only in the seasonally dry forests of the inter-Andean valleys of the Marañon river and its tributary, the Utcubamba, with the exception of one species (Meerow and Nakamura, 2019), is a xerophyte with thick, carnose leaves and universally green (or green and white) flowers. Floral morphology is similar to both *Phaedranassa* [*R. multiflora* (Kunth) Ravenna, *R. staminosa* Ravenna] and *Eucrosia* (*R. decora* Ravenna).

The next branch is the monotypic *Plagiolirion horsmannii*, endemic to the Cauca valley of Colombia, and possibly of reticulate origin (Figure 7). This relict genus, most recently allied with *Eucharis* (Meerow and Silverstone-Sopkin, 1995), is a clear case of peripheral isolation. It adapted to lower and mid-montane rainforest understory of the Cauca valley, exhibiting some of the characters of *Eucharis* (thin-textured leaf laminae, ellipsoidal seeds with copious oily endosperm), which we interpret as convergences. Plastome sequences position it as sister to *Eucrosia* and *Phaedranassa* (Figure 4).

The sister relationship of *Eucrosia* and *Phaedranassa* is ironclad, and their divergence at ca. 10 Mya may have been directly influenced by the rise of the Andes. *Phaedranassa* is primarily found above 2,000 m in the Ecuadorian Andes, which is not evident by the breadth of our sampling (only a single Ecuadorian species of eight was included), while *Eucrosia* is markedly a low elevation genus, rarely found above 1,000 m. The leaves of *Eucrosia* have a smaller length:width ratio than its sister genus, and the perigones are zygomorphic [though not strongly so in *Eucrosia stricklandii* (Baker) Meerow] vs. the actinomorphy of *Phaedranassa*. Most importantly, all true *Eucrosia* spp. have globose nectar glands at the base of the stamens (Meerow, 1987a). Like its sister genus, most of its species diversity is found in Ecuador (Meerow, 1990), but unlike *Phaedranassa*, which does not occur south of Loja Province, *Eucrosia* is represented in Peru by one endemic [*Eucrosia calendulina* Meerow and Sagástegui (1997)] and two species that also occur in Ecuador (*Eucrosia bicolor*, albeit a distinct variety, and *Eucrosia eucrosioides* Pax). Only two species, *E. bicolor* and *E. stricklandii*, have a conspicuous staminal cup that is deeply cleft dorsally (Meerow, 1987a).

The final and somewhat problematic subclade in the Eucharideae resolved a monophyletic *Stenomesson*, inclusive of *Caliphruria korsakoffii*^4^, the only Peruvian species of that genus. This species was the first branch in *Stenomesson* (Figures 2, 3), and may represent an isolated eastern relict of early diversification of the subclade (Figure 7), as it is known only from wet forest in the vicinity of Moyobamba, Peru (Meerow, 1989). The two species of *Stenomesson* that release their pollen in tetrads (Meerow et al., 1986; Meerow and van der Werff, 2004), *S. chloranthum* Meerow & van der Werff and *S. leucanthum*, (Ravenna) Meerow & van der Werff, were sister species in every tree generated (Figures 2–4 and Supplementary Figures 2–7). Ravenna (1988) *Stenomesson* sect. *Adnata* Ravenna, in which the staminal cup is almost completely fused to the perigone tube, represented in our sampling by *S. ecuadorense* and *S. miniatum*, was resolved as monophyletic by the full nuclear gene supermatrix and all of the species trees, but not the 70–90% supermatrix trees nor the plastome supermatrix.

The sister clade to *Stenomesson* is completely adapted to the shady understory of low to mid-elevation rainforest, and extends the furthest east of any taxa in the tribe (Meerow, 1989). It showed a high degree of reticulation in the hybridization network (Figure 7). In this rainforest understory clade, the Colombian *Caliphruria* spp. were nested in a paraphyletic *Eucharis* (Figures 2, 3) that was sister to *Urceolina*. *Eucharis amazonica* and *E. sanderi*, which Meerow (1989) placed in subg. *Heterocharis* Meerow along with *Eucharis moorei* (Baker) Meerow and one natural hybrid, and which he considered the least derived species in the genus, constituted the first branch after *Urceolina* (Figures 2, 3). The most intriguing subclade brought together *E. dodsonii*, *E. astrophiala*, and the Colombian *Caliphruria* spp. (Figures 2, 3). *Eucharis astrophiala* is the only species of the genus endemic to the lower western Andean slopes of Ecuador, and bears novel morphological characteristics suggestive of peripheral isolation (Meerow, 1989). Like its sister species, *E. dodsonii*, also endemic to the western Ecuadorean mid-elevation (900–1,500 m) montane rainforest, it has deeply plicate leaves (Meerow and Dehgan, 1985). This small subclade of four species (Figures 2, 3 and Supplementary Figures 3,4) dramatically illustrates the convergent evolutionary machinations of isolation on the tetraploid genomes of the Andean clades (Meerow, 2010). *E. dodsonii*, misdiagnosed as a *Eucrosia* because of its long exerted stamens, despite its mesic ecology (Meerow and Dehgan, 1985) and small globose seeds (unknown at the time it was described), is analogous to the divergence of *Urceolina* from *Eucharis*, and even bears similar perigonal coloration as the former. Although he was wrong in regard to *Mathieua* and *Plagiolirion* (Meerow, 1987d; Meerow and Silverstone-Sopkin, 1995), we recognize the prescience of Traub (1971) in combining *Caliphruria* (less *C. korsakoffii*), *Eucharis* and *Urceolina* as the latter, and accept this clade as a single genus, *Urceolina*, now with the addition of *E. dodsonii*^5^ (Figures 2–4, 7).

### Did the Amotape—Huancabamba Zone (AHZ) Function as a Barrier in the Early Diversification of Eucharideae?

The northern and central Andes connect in southern Ecuador and northern Peru, and this area does not achieve elevations above 2,145 m (Weigend, 2002). This depression in the Andes was labeled as the Amotope–Huancabamba Zone (AHZ) by Berry (1982), and was discussed in greater detail by Weigend (2002, 2004). It has also been called the Huancabamba deflection, depression or gap (Quintana et al., 2017b). It is the youngest part of the Andean chain (Hoorn et al., 2010).

The AHZ was invaded by marine incursions in the Eocene (see discussion in the next section), and has been considered a dispersal barrier for lowland taxa from west to east or vice versa (Antonelli et al., 2009). As the Andes rose, these marine incursions ended. Subsequently, the AHZ was considered by some authors to have become a more recent barrier for upland plant taxa in a north/south (and reverse) direction on the basis of biogeographic study (Ayers, 1999; Beck and Richter, 2008; Antonelli et al., 2009; Cosacov Martinez et al., 2009; Antonelli and Sanmartín, 2011; Quintana et al., 2017b), despite previous contrary conclusions (Berry, 1982; Weigend, 2002). Quintana et al. (2017b) concluded that the AHZ functioned as a migration corridor for taxa both in latitudinal and longitudinal directions, but not as a barrier. Mutke et al. (2014) also strongly rejected the barrier hypothesis, and point out the high degree of plant diversity in the zone.

We believe that the distributions of the first branches in the Eucharideae clade suggest that the AHZ may have functioned both as an east-west and north-south corridor, as well as a north-south barrier to migration. The first branch of the tribe is the xerophytic genus *Rauhia*, the five species of which are found only at the southern limits of the AHZ (Meerow and Nakamura, 2019), generally at 500–800 m elevation. The next branch, *Plagiolirion*, is endemic to Colombia, but may be of recombinant origin (Figure 7), or perhaps another instance of ILS. It is impossible to say if it represents a long distance dispersal event (Figure 10), or an element of a once more widespread lineage of rainforest understory taxa. The next subclade, sister genera *Eucrosia* and *Phaedranassa*, are lowland and highland elements, respectively, the diversity of which is concentrated in Ecuador (Meerow, 1990). *Phaedranassa* is not known south of Loja Province in Ecuador at the northern limits of the AHZ (Meerow, 1990), while *Eucrosia* has managed to migrate west of the Andes to the Pacific coastal plain (*E. bicolor*), and south into Peru [*E. bicolor* var. *plowmanii* Meerow, *E. eucrosioides* (Meerow, 1987a) and the endemic *E. calendulina* (Meerow and Sagástegui, 1997)].

### Additional Biogeographical and Historical Inferences and Caveats

In calibrated age diversification studies of woody plant lineages from seasonally dry Andean tropical forests, splits between clades dating from the Miocene to Pliocene have been observed with strong geographic structure (Pennington et al., 2004, 2010; Särkinen et al., 2012; Lavin et al., 2018), attributed to speciation via enforced geographic isolation (Pennington et al., 2009). This certainly seems to be the case in many of the genera of the Andean tetraploid clade, in which closely related species are found in isolated inter-Andean valleys, e.g., *Rauhia* (Meerow and Nakamura, 2019), *Eucrosia* (Meerow, 1987a) and *Phaedranassa* (Oleas et al., 2012, 2013, 2016) over relatively short distances.

Though the exact timing and rate of Andean uplift remains controversial (Garzione et al., 2008; Picard et al., 2008; Antonelli et al., 2009; Luebert and Weigend, 2014; Mutke et al., 2014), there is greater consensus on the ways in which the vast and heterogeneous orogenic chain has directly affected plant diversification (Simpson, 1975, 1983; Antonelli and Sanmartín, 2011; Hoorn et al., 2013; Luebert and Weigend, 2014). The main classes of effects are (1) creation of new high elevation habitats, (2) providing barriers that result in vicariance (Figure 10), (3) providing a bi-directional north-south migration corridor, and (4) generating new environments for colonization outside of the Andes. All of these seem to have contributed to the diversification of the Andean clade of Amaryllidaceae. *Phaedranassa* is a good example of the first. The second and third are significantly broad effects on the entire clade (Figure 10). Lastly, the divergence of *Hymenocallis* from its Andean roots might be an example of the fourth.

From the Eocene to the Middle Miocene, there appear to have been incursions from the Pacific that flooded a low elevation area between the Northern and Central Andes in the vicinity of southern Ecuador/northern Peru, known as the “Western Andean Portal” (WAP) or “‘Guayaquil Gap”’ (Hoorn, 1993; Hoorn et al., 1995; Steinmann et al., 1999; Santos et al., 2008; Antonelli et al., 2009). The WAP has been cited as an important barrier that split lineages of various biota (Molau, 1988; Sparre and Andersson, 1991; Hofreiter and Rodríguez, 2006; Antonelli et al., 2009; Meerow et al., 2015). We believe that this may have been a contributing factor for the relative dearth of species of both *Clinanthus* and *Stenomesson* north of Peru.

The flooding of western Amazonia caused by the uplift of the Eastern Cordillera of the Central Andes in the early Miocene and subsequent Caribbean marine incursion from the north (Lundberg et al., 1998; Wesselingh et al., 2002; Wesselingh, 2006; Wesselingh and Salo, 2006; Antonelli et al., 2009; Meerow et al., 2009, 2014; Roncal et al., 2013), created an enormous system of long-lived lakes and wetlands (“Lake Pebas” or the “Pebas Sea”) that provided a significant barrier to dispersal between the eastern Amazonian and Guianan region and the Andes lasting until the Late Miocene. We suggest that this event isolated *Hymenocallis* from its ancestors in the Andean region.

Age of these deeper internal nodes in our penalized likelihood age divergence tree (Figure 9) should be approached with caution in the absence of fossil calibration, especially since in Bayesian age diversification analyses, it is the deeper branches in phylogenetic trees that typically show the broadest 95% highest prior densities (e.g., Meerow et al., 2009, 2015; Salas-Leiva et al., 2013; Calonje et al., 2019). Consequently, considering that the deeper calibration is based on a previous chronogram from analysis of a few plastid regions, and only four American species (Santos-Gally et al., 2012), an Oligocene origin for the clade might be an overestimation of its age. However, the other calibration point is based on a solid basis of studies with tree fossil support of a minimum age for the type of inter-Andean dry forests in which the genus *Rauhia* is endemic (Caetano et al., 2008; Pennington et al., 2010; Luebert and Weigend, 2014; Quintana et al., 2017a). Consequently, the node ages above the stem of *Rauhia* should have greater veracity.

A final caveat is one that needs to be at least acknowledged. Before Europeans arrived on South American shores, successive human cultures inhabited the Andean region, and the final one, the Inca, though relatively short-lived, ruled an empire that stretched from Ecuador to southern Peru (Burger, 1995). The amaryllis subfamily of Amaryllidaceae produce beautiful flowers, and its members contain many novel alkaloids with biological properties. Ceremonial drinking vessels (keros) from the Incan culture bear iconic floral depictions of the species *C. humilis*, *S. miniatum* and perhaps a *Pyrolirion* sp. (Vargas, 1962). A festival celebrating the June gregarious flowering of *Ismene amancaes* Herb. on the *lomas* near Lima that lasted until the middle of the 19th century may have predated the arrival of Europeans (Stewart, 1832). Remains of the plant have been found in Peruvian archeological sites (Browman, 1978). *S. pearcei*, which has generally been thought to occur only in the mountains above Cusco (Macbride, 1936), was recently found in five populations in the Kañaris District, Lambayeque Department between 2,600 and 3,000 m (B. Esquerre, pers. comm), perhaps an anthropogenically facilitated disjunction. Northern Peru is where the greatest diversity of *Stenomesson* diversity can be found (Meerow, 1987b; Meerow and van der Werff, 2004). The possible influence of humans on the modern range of species in the Andean clade, though difficult to document, should not be discounted.

## Conclusion

The Andean tetraploid clade of the Amaryllidaceae subfam. Amaryllidoideae has been characterized as a highly canalized complex suffused with convergent patterns of divergence within the four tribal lineages (Meerow, 2010). This is not merely a matter of taxonomic judgment, but reflects the evolutionary forces at work since its polyploid origin. An association between polyploidy and both morphological and ecological divergence has long been noted (Ohno, 1970; Levin, 1983), but in the genomic age, our understanding of the degree to which polyploidy fuels evolutionary change in plants has become more explicit (Adams and Wendel, 2005; Soltis et al., 2015). Polyploids may undergo fast and sweeping genomic changes (Lynch and Conery, 2000; Seoighe and Gehring, 2004). Evidence has accumulated showing that duplicate genes either diversify in function or undergo subfunctionalization (Lynch and Force, 2000; Blanc and Wolfe, 2004). Polyploidy also effects gene expression, including gene silencing (Kashkush et al., 2002; Adams et al., 2003; He et al., 2003).

Of the external selective forces operating on the polyploid genome of the Andean clade, the most obvious is the rise of the Andes itself, an association that has been noted repeatedly (Meerow and Dehgan, 1985; Meerow, 1987c,d, 2010) and is obvious from our biogeographic analyses (Figure 10 and Supplementary Figure 9). Meerow (2010) characterized the Andean tetraploid clade’s polyploid genome as grist for the mill of the Andean orogeny. Northern Peru in particular, comprising in part the Neotropical bioprovinces Cauca, Desert and Ecuadorian (Morrone, 2014), with its complex of microhabitats, has been a hotspot of diversification in the Andean clade (Meerow and van der Werff, 2004; Meerow and Cano, 2019; Meerow and Nakamura, 2019; Esquerre and Meerow, 2020). The distributions of *Rauhia* in Peru and *Phaedranassa* in Ecuador (Meerow, 1990; Oleas et al., 2012, 2013, 2016) fit the allopatric models of dispersal or vicariance (Figure 10 and Supplementary Figure 9) followed by isolation described for Andean complexes of *Buddleja* L. (Norman, 2000), *Calceolaria* L. (Molau, 1988), *Fuchsia* L. (Berry, 1982), and many other taxa (Ayers, 1999; Beck and Richter, 2008; Antonelli et al., 2009; Pennington et al., 2010; Anthelme et al., 2014; Luebert and Weigend, 2014; Bacon et al., 2018).

Compared to the tribe Hippeastreae (Meerow, 2010; García et al., 2014), reticulation has not played a significant role in the generic radiation of the Andean tetraploid clade, except in Eucharideae, where we must concede that *Eucharis* and Colombian *Caliphruria* cannot be logically separated from *Urceolina* (Traub, 1971). However, there is evidence of large scale interspecific hybridization within *Hymenocallis* in the SE U.S. and Mexico, and to a lesser extent in *Clinanthus*. We only see ILS as a significant factor in the genus *Pamianthe*, and perhaps *Plagiolirion* as well.

In the context of the four hypotheses we tested, (1) phylogenomic data support the previous tribal and generic classification of the tetraploid Andean clade inferred from ITS and several plastid loci, (2) reticulation was primarily limited to interspecific gene flow at the generic level, with one notable exception in the Eucharideae, (3) the Andean orogeny was the primary factor in the generic diversification of the clade and (4) our resolute phylogeny suggest that polyploidy and resulting paralogy do not necessarily impede accurate phylogenomic inference.

It may be tempting for some taxonomic pundits to declare that there has been superfluous recognition of genera within the Andean clade, and suggest further mergers beyond our acceptance of Traub (1971) concept of *Urceolina*. We believe that would be an error of judgment that obscures the phylogenetic complexity of this Andean-centered polyploid radiation, could result in loss of information, confusion, and would create further nomenclatural chaos in a family already rife with it (García et al., 2019).

## Data Availability Statement

Illumina HiSeq reads (fastq files) and associated information from the sequence capture are deposited in the Sequence Read Archive (SRA) database of The National Center for Biotechnology Information as Bioproject PRJNA635412. Alignments, analysis data files and trees are deposited in DRYAD Project: doi: 10.5061/dryad.573n5tb4j.

## Author Contributions

AM and KN designed the study. KN conducted all lab work at USDA prior to the contracted services from Rapid genomics, and assembled the plastome data. AM and EG conducted all analyses. AM, EG, and KN wrote the manuscript. All authors read and approved the manuscript.

## Conflict of Interest

The authors declare that the research was conducted in the absence of any commercial or financial relationships that could be construed as a potential conflict of interest.

## Footnotes

1 https://www.bioinformatics.babraham.ac.uk/projects/trim_galore/

2 https://www.geneious.com

3 *Hymenocallis gigantiflora* Meerow, nom. nov. See Supplementary Text 1 for full details.

4 *Stenomesson korsakoffii* (Traub) Meerow, comb. nov. See Supplementary Text 1 for full details.

5 *Urceolina dodsonii* (Meerow & Dehgan) Meerow, comb. nov. See Supplementary Text 1 for full details.

## Supplementary Material

Supplementary Text 1 Taxonomic changes.

Supplementary Figure 1 Heat map of paralog presence across the unpruned sequence capture loci from 95 species of Andean Amaryllidaceae and 5 outgroups. White = absent, black = one copy, yellow-to-red = 2 to 11 copies (paralogs).

Supplementary Figure 2 Best tree from maximum likelihood analysis of partitioned 524 nuclear genes, coding-only supermatrix of the Andean tetraploid clade of Amaryllidaceae subfam. Amaryllidoideae, with bootstrap percentages < 100 shown above branches (all unmarked branches have 100% BP). Refer to Supplementary Table 1 for corrected taxon names.

Supplementary Figure 3 Best tree from maximum likelihood analysis of partitioned 260 nuclear gene supermatrix (only genes amplified across 70% of the Andean tetraploid clade of Amaryllidaceae subfam. Amaryllidoideae), with bootstrap percentages < 100 shown above branches (all unmarked branches have 100% BP). Refer to Supplementary Table 1 for corrected taxon names.

Supplementary Figure 4 Best tree from maximum likelihood analysis of partitioned 137 nuclear gene supermatrix (only genes amplified across 90% of the Andean tetraploid clade of Amaryllidaceae subfam. Amaryllidoideae), with bootstrap percentages < 100 shown above branches (all unmarked branches have 100% BP).

Supplementary Figure 5 Maximum likelihood tree with bootstrap using an earlier complete supermatrix of the Andean tetraploid clade of Amaryllidaceae in which consensus sequences of all paralogs with ambiguity codes were used.

Supplementary Figure 6 Local posterior probability (LPP) coalescent species tree from ASTRAL III analysis of gene trees from 260 nuclear genes with 70% taxon coverage. LPP scores appear above the branches; bootstrap percentages < 100 shown below branches (all unmarked branches have 100% BP). Refer to Supplementary Table 1 for corrected taxon names.

Supplementary Figure 7 Local posterior probability (LPP) coalescent species tree from ASTRAL III analysis of gene trees from 70 nuclear genes with 90% taxon coverage. LPP scores appear above the branches; bootstrap percentages < 100 shown below branches (all unmarked branches have 100% BP). Refer to Supplementary Table 1 for corrected taxon names.

Supplementary Figure 8 Results of the densiTree comparison of relaxed and correlated penalized likelihood age-calibration of the best maximum likelihood tree found by RAxML using the 90% taxon coverage supermatrix and a range of values for λ (0–5).

Supplementary Figure 9 Area cladogram generated by Bayarealike +J analysis with BioGeoBears through RASP 4.2, using the best maximum likelihood tree found for the partial plastome supermatrix. Areas at any node were limited to three, and only the most likely was mapped onto the tree.

Supplementary Table 1 Samples used in the phylogenomic analyses of the Andean tetraploid clade of Amaryllidaceae subfam. Amaryllidoideae, and accession numbers for fastq files from the sequence capture.

Supplementary Table 2 Nuclear gene alignments generated from anchored hybrid sequence capture of 100 taxa of the of Amaryllidaceae subfam. Amaryllidoideae.

Supplementary Table 3 Event tally for DIVALIKE+J biogeographic analysis of the best 90% taxon coverage supermatrix tree found by maximum likelihood. Node numbers refer to Figure 10.

Supplementary Table 4 Event tally for BAYAREALIKE +J biogeographic analysis of the partial plastome supermatrix best ML tree. Node numbers refer to Supplementary Figure 10.

Supplementary Table 5 Pseudogenization of *ndh* genes in plastomes of Eucharideae.
