# Corrigendum: Phylogenomics of the Andean tetraploid clade of the American Amaryllidaceae (subfamily Amaryllidoideae): Unlocking a polyploid generic radiation abetted by continental geodynamics

**DOI:** 10.3389/fpls.2023.1151864

**Published:** 2023-03-02

**Authors:** Alan W. Meerow, Elliot M. Gardner, Kyoko Nakamura

**Affiliations:** ^1^ USDA-ARS-SHRS, National Clonal Germplasm Repository, Miami, FL, United States; ^2^ Singapore Botanic Gardens, National Parks Board, Singapore, Singapore; ^3^ Institute of Environment, Florida International University, Miami, FL, United States

**Keywords:** anchored hybrid enrichment, Andes, Asparagales, biogeography, geophyte, molecular systematics, monocotyledons, phylogenetics

In the published article, there was an error in **Supplementary Text 1**. The file (**Supplementary Datasheet 10**) containing taxonomic changes was uploaded in Word format (.docx). This violates Art. 29.1 of the International Code for Botanical Nomenclature for effective publication. In order for the new botanical names to be duly registered, the Word file has been replaced by a PDF file. There was no error in the file text. The new file for **Supplementary Text 1** in the correct format appears below.

## Supplementary material

SUPPLEMENTARY TEXT 1 Taxonomic changes.

