# Supplementary material for: Phylogenomics of the Andean Tetraploid Clade of the American Amaryllidaceae (Subfamily Amaryllidoideae): Unlocking a Polyploid Generic Radiation Abetted by Continental Geodynamics
Source: Front Plant Sci. 2020 Nov 5;11:582422. doi: 10.3389/fpls.2020.582422 (PMC7674842; doi:10.3389/fpls.2020.582422)
Supplement: Supplementary Text 1 — Taxonomic changes. [file Data_Sheet_10.docx]

**Supplemental Text S1. TAXONOMIC CHANGES**

**Eucharideae**

Nicholson (1884) transferred C. *hartwegiana* Herb. (the type species of *Caliphruria* Herb.) to *Eucharis,* ignoring the former's nomenclatural priority. Traub (1967) made the formal transfer of the remaining species of *Caliphruria,* C. *tenera* Baker, to *Eucharis* [*Caliphruria subedentata* Baker had been previously been transferred to *Eucharis* by Bentham and Hooker (1883)] and combined the monotypic *Plagiolirion* Baker with *Eucharis.* He had previously listed *Caliphruria, Plagiolirion,* and *Mathieua* Klotzsch (another monotypic genus) as synonyms for *Eucharis* in his *Genera of the Amaryllidaceae* (Traub, 1963)*,* citing Baillon (1894) as a "special reference." The nomenclatural priority of *Caliphruria* Herbert was overlooked again. No proposal for the conservation of *Eucharis* over *Caliphruria* has ever been proposed previous to that of Meerow & Dehgan (1984). Traub (1971) later combined *Eucharis* with *Urceolina.* He designated five subgenera: *Urceolina, Eucharis, Caliphruria, Mathieua,* and *Plagiolirion.* Traub (1971) provided no explanation for the combination, but presumably his decision was prompted in part by reports in the literature of two intergeneric hybrids between *Eucharis* and *Urceolina:* ×*Urceocharis clibranii* Masters (1892), an artificial hybrid, and ×*U. edentata* C. H. Wright (1910), putatively discovered in Peru. Meerow (1989) also recognized a putative intergeneric hybrid between *Eucharis* and *Caliphruria* as ×*Calicharis butcheri* (Traub) Meerow*.*

As discussed in the main text of this paper, a new combination in *Urceolina* is necessary for *Eucrosia* *dodsonii*.

*Urceolina dodsonii* (Meerow & Dehgan) **Meerow**, **comb. nov.**

*Eucrosia dodsonii* Meerow & Dehgan (1985). *Brittonia* 37: 47-55.

**Type:** Ecuador, Cotopaxi: Tenefuerste, Río Pilalo, Km 52-53 Quevido- Latacunga, 750-900 m, common, in mature forest, 19 Jul 1982, *Dodson & Embree 13451* (holotype: MO!; isotype: SEL!).

Finally, a new combination is *Stenomesson* is necessary for the former *Eucharis* *korsakoffii*:

*Stenomesson korsakoffii* (Traub) **Meerow**, **comb. nov.**

*Eucharis korsakoffii* Traub (1967), *Pl. Life* (Stanford) 23: 85 (-87).

*Urceolina korsakoffi* (Traub) Traub (1971), *Pl. Life* (Stanford) 27: 59.

*Caliphruria korsakoffi* (Traub) Meerow (1989), *Ann. Missouri Bot. Gard.* 76: 217.

**Type:** Peru, San Martín: 40 km from Moyobamba, Hierra waterfalls, 1,500 m, ex hort. J. N. Giridlian from bulbs collected by Lee Moore, 16 July 1966, *Traub 1060* (holotype, MO!).

**Hymenocallideae**

Standley and Steyermark (1944) described a novel species of *Hymenocallis* from eastern Guatemala growing in rainforest understory as *Pancratium guatemalense* Standl. & Steyerm.*,* which they did not differentiate from *Hymenocallis*. This species has never been properly transferred to *Hymenocallis*. A few years ago we received living material of a Guatemalan *Hymenocallis* from a location very close to the type locality [Guatemala, Prov. Izabel, Rio Las Escabas, west of Puerto Barrios, *Meerow 3610* (NA)]. The plant produces very large flowers and has striking long and wide pseudopetiolate leaves. When we reviewed photographs of the holotype and paratype of *P. guatemalense*, it was clear that our plant was conspecific. As the epiphet *guatemalensis* is preoccupied in *Hymenocallis* by *H. guatemalensis* Traub (1967) from a distant locality and different habitat in the country, a new name for the transfer is necessary. This species bears the longest flowers of any species in the genus.

*Hymenocallis gigantiflora* **Meerow**, **nom. nov.**

*Pancratium guatemalense* Standl. & Steyerm. (1944). Publ. Field Mus. Nat. Hist., Bot. Ser. 23: 38. Non *H. guatemalensis* Traub (1967).

**Type:** Cultivated at Garfield Park Conservatory, Chicago, flowering in November; bulbs collected 13 April 1940 in Guatemala, Dept. Izabal (along stream near waterfall between Escobas and Montana Escobas, across the bay from Puerto Barrios, at or near sea level), 5 November 1941, *Steyermark* *39865* (holotype**:** F, photo!); *Steyermark 39288* (paratype; F, photo!).

**REFERENCES**

Baillon, M.H. (1894). Sur les limites du genre *Caliphruria*. *Bull. Mens. Soc. Linn. Paris* 144, 1133-1136.

Bentham, G. & Hooker, J.D. (1883). *Eucharis subedentata* (Baker) Benth. & Hook. f. *Gen. Pl.* 3(2), 731.

Masters, T.M. (1892). x*Urceocharis* *clibranii*. *Gard. Chron. ser. 3* 12, 214, Figure 36.

Meerow, A.W., and Dehgan, B. (1984). Proposal to conserve 1196 *Eucharis* against *Caliphruria* (Amaryllidaceae). *Taxon* 33, 516-517. [doi: 10.2307/1220998](https://doi.org/10.2307/1220998)

Nicholson, G. (1884). *Eucharis hartwegiana* (Herb.) Nicholson. *Ill. Dict. Gard.* 1, 536.

Standley, P.A. and Steyermark, J.F. (1944). *Pancratium guatemalense* Standl. & Steyerm. (1944). *Publ. Field Mus. Nat. Hist., Bot. Ser.* 23, 38.

Traub, H.P. (1963). *Genera of the Amaryllidaceae*. American Plant Life Society. La Jolla, California.

Traub, H.P. (1967). Amaryllid notes. *PI. Life* 23, 65.

Traub, H.P. (1967). *Hymenocallis guatemalensis* Traub, *Pl. Life* 23, 67.

Traub, H.P. (1971). Amaryllid notes. *PI. Life* 27, 57-59.

Wright, C.H. (1910). ×*Urceocharis edentata.* *Bull. Misc. Inform. Kew* 1, 24.
