# Supplementary material for: Phylogenomics of the Andean Tetraploid Clade of the American Amaryllidaceae (Subfamily Amaryllidoideae): Unlocking a Polyploid Generic Radiation Abetted by Continental Geodynamics
Source: Front Plant Sci. 2020 Nov 5;11:582422. doi: 10.3389/fpls.2020.582422 (PMC7674842; doi:10.3389/fpls.2020.582422)
Supplement: Supplementary Figure 1 — Heat map of paralog presence across the unpruned sequence capture loci from 95 species of Andean Amaryllidaceae and 5 outgroups. White = absent, black = one copy, yellow-to-red = 2 to 11 copies (paralogs). [file Data_Sheet_1.PDF]

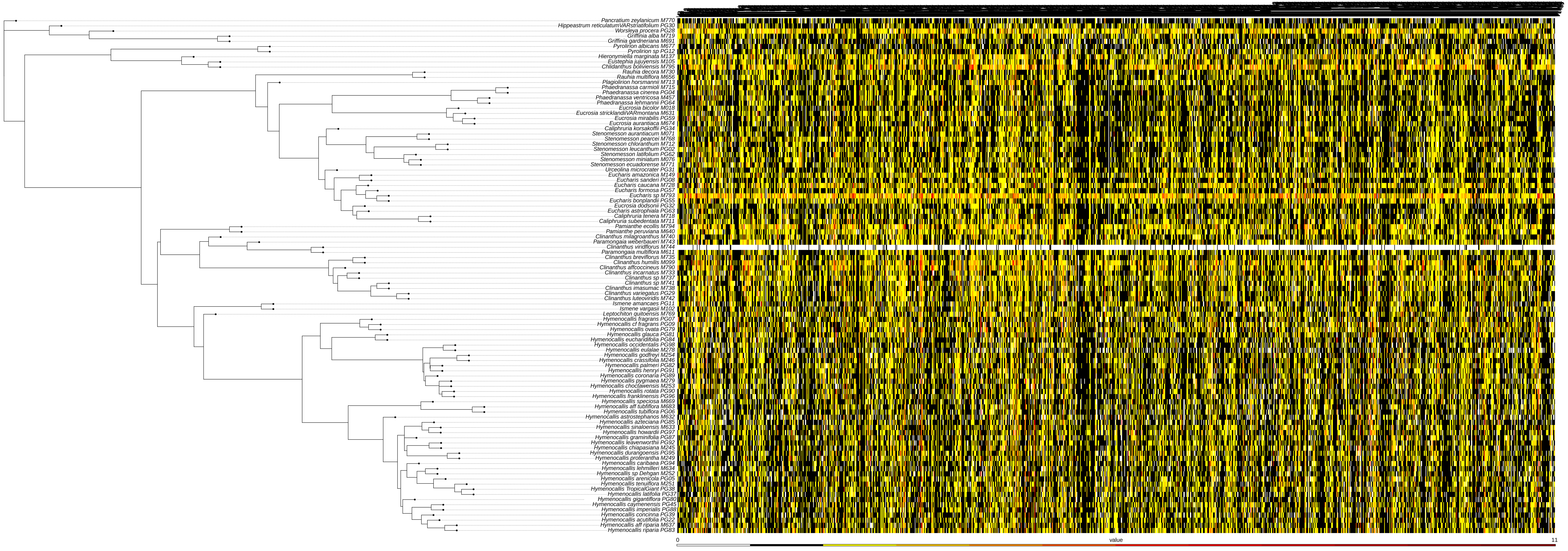

Pancratium zeylanicum M770
Hippeastrum reticulatumVARstriatifolium PG30
Worsleya procera PG28
Griffinia alba M719
Griffinia gardneriana M691
Pyrolirion albicans M677
Pyrolirion sp PG12
Hieronymiella marginata M137
Eustephia jujuyensis M105
Chlidanthus boliviensis M795
Rauhia decora M730
Rauhia multiflora M656
Plagiolirion horsmannii M713
Phaedranassa carmioli M715
Phaedranassa cinerea PG04
Phaedranassa ventricosa M457
Phaedranassa lehmannii PG64
Eucrosia bicolor M018
Eucrosia stricklandiiVARmontana M631
Eucrosia mirabilis PG59
Eucrosia aurantiaca M674
Caliphruria korsakoffii PG34
Stenomesson aurantiacum M071
Stenomesson pearcei M768
Stenomesson chloranthum M712
Stenomesson leucanthum PG02
Stenomesson latifolium PG62
Stenomesson miniatum M076
Stenomesson ecuadorense M771
Urceolina microcrater PG31
Eucharis amazonica M149
Eucharis sanderi PG08
Eucharis caucana M728
Eucharis formosa PG57
Eucharis sp M793
Eucharis bonplandii PG55
Eucrosia dodsonii PG32
Eucharis astrophiala PG63
Caliphruria tenera M718
Caliphruria subedentata M711
Pamianthe ecollis M794
Pamianthe peruviana M640
Clinanthus milagroanthus M740
Paramongaia weberbaueri M743
Clinanthus viridflorus M744
Paramongaia multiflora M611
Clinanthus breviflorus M735
Clinanthus humilis M099
Clinanthus affcoccineus M790
Clinanthus incarnatus M733
Clinanthus sp M737
Clinanthus sp M741
Clinanthus imasumac M738
Clinanthus variegatus PG29
Clinanthus luteoviridis M742
Ismene amancaes PG11
Ismene vargasii M102
Leptochiton quitoensis M769
Hymenocallis fragrans PG07
Hymenocallis cf fragrans PG09
Hymenocallis ovata PG79
Hymenocallis glauca PG81
Hymenocallis eucharidifolia PG84
Hymenocallis occidentalis PG98
Hymenocallis eulalae M278
Hymenocallis godfreyi M254
Hymenocallis crassifolia M246
Hymenocallis palmeri PG82
Hymenocallis henryi PG91
Hymenocallis coronaria PG89
Hymenocallis pygmaea M279
Hymenocallis choctawensis M253
Hymenocallis rotata PG90
Hymenocallis franklinensis PG96
Hymenocallis speciosa M669
Hymenocallis aff tubfiflora M683
Hymenocallis tubiflora PG06
Hymenocallis astrostephanos M632
Hymenocallis azteciana PG85
Hymenocallis sinaloensis M633
Hymenocallis howardii PG97
Hymenocallis graminifolia PG87
Hymenocallis leavenworthii PG92
Hymenocallis chiapasiana M245
Hymenocallis durangoensis PG95
Hymenocallis proterantha M249
Hymenocallis caribaea PG94
Hymenocallis lehmilleri M634
Hymenocallis sp Dehgan M252
Hymenocallis arenicola PG05
Hymenocallis tenuiflora M251
Hymenocallis TropicalGiant PG38
Hymenocallis latifolia PG37
Hymenocallis gigantiflora PG80
Hymenocallis caymenensis PG45
Hymenocallis imperialis PG88
Hymenocallis concinna PG39
Hymenocallis acutifolia PG22
Hymenocallis aff riparia M637
Hymenocallis riparia PG83
0
value
11
