# Supplementary figures and images for: Phylogenomics of the Andean Tetraploid Clade of the American Amaryllidaceae (Subfamily Amaryllidoideae): Unlocking a Polyploid Generic Radiation Abetted by Continental Geodynamics
Source: Front Plant Sci. 2020 Nov 5;11:582422. doi: 10.3389/fpls.2020.582422 (PMC7674842; doi:10.3389/fpls.2020.582422)

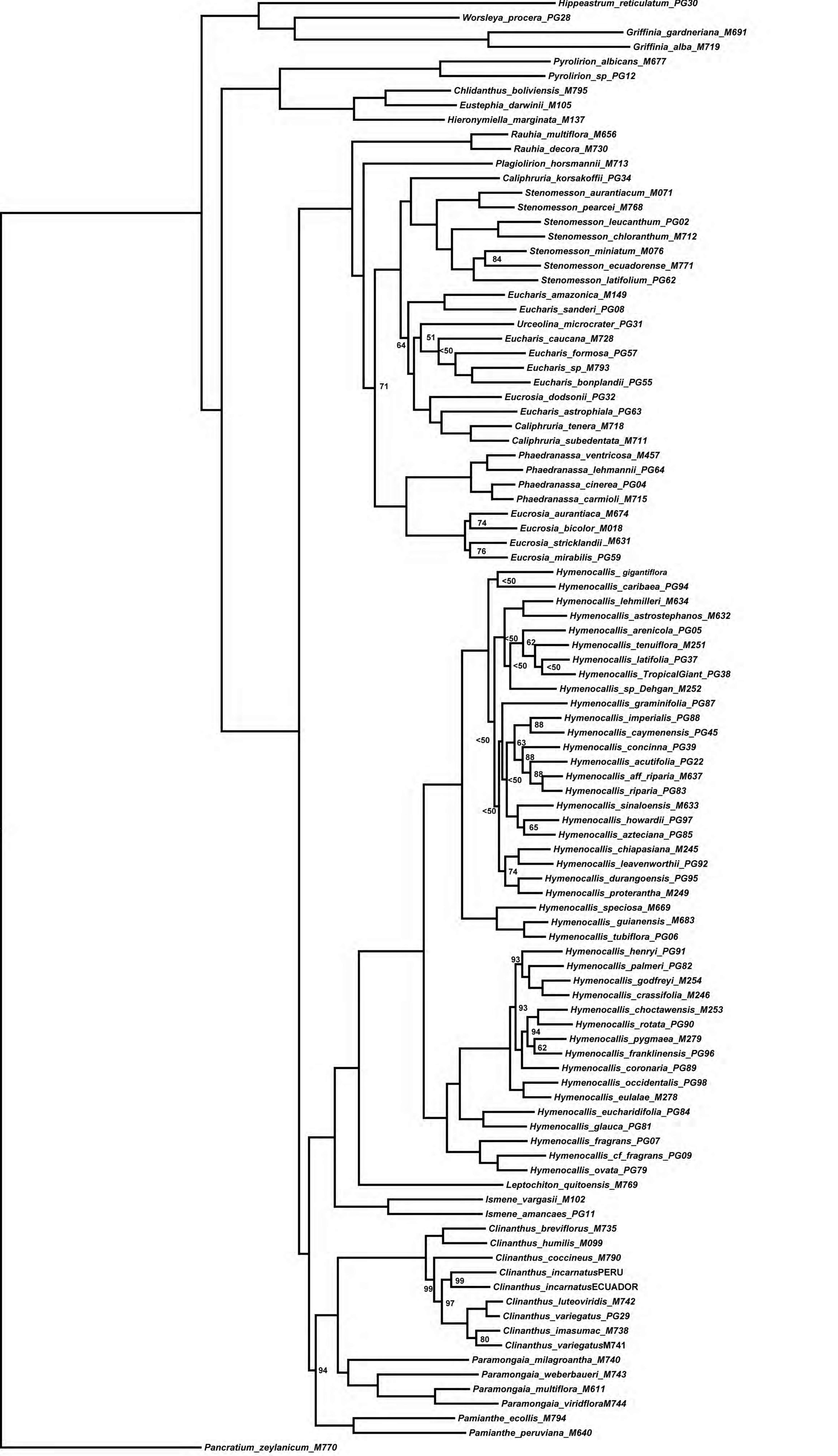

Supplement: Supplementary Figure 2 — Best tree from maximum likelihood analysis of partitioned 524 nuclear genes, coding-only supermatrix of the Andean tetraploid clade of Amaryllidaceae subfam. Amaryllidoideae, with bootstrap percentages < 100 shown above branches (all unmarked branches have 100% BP). Refer to Supplementary Table 1 for corrected taxon names. [file Data_Sheet_2.PDF]

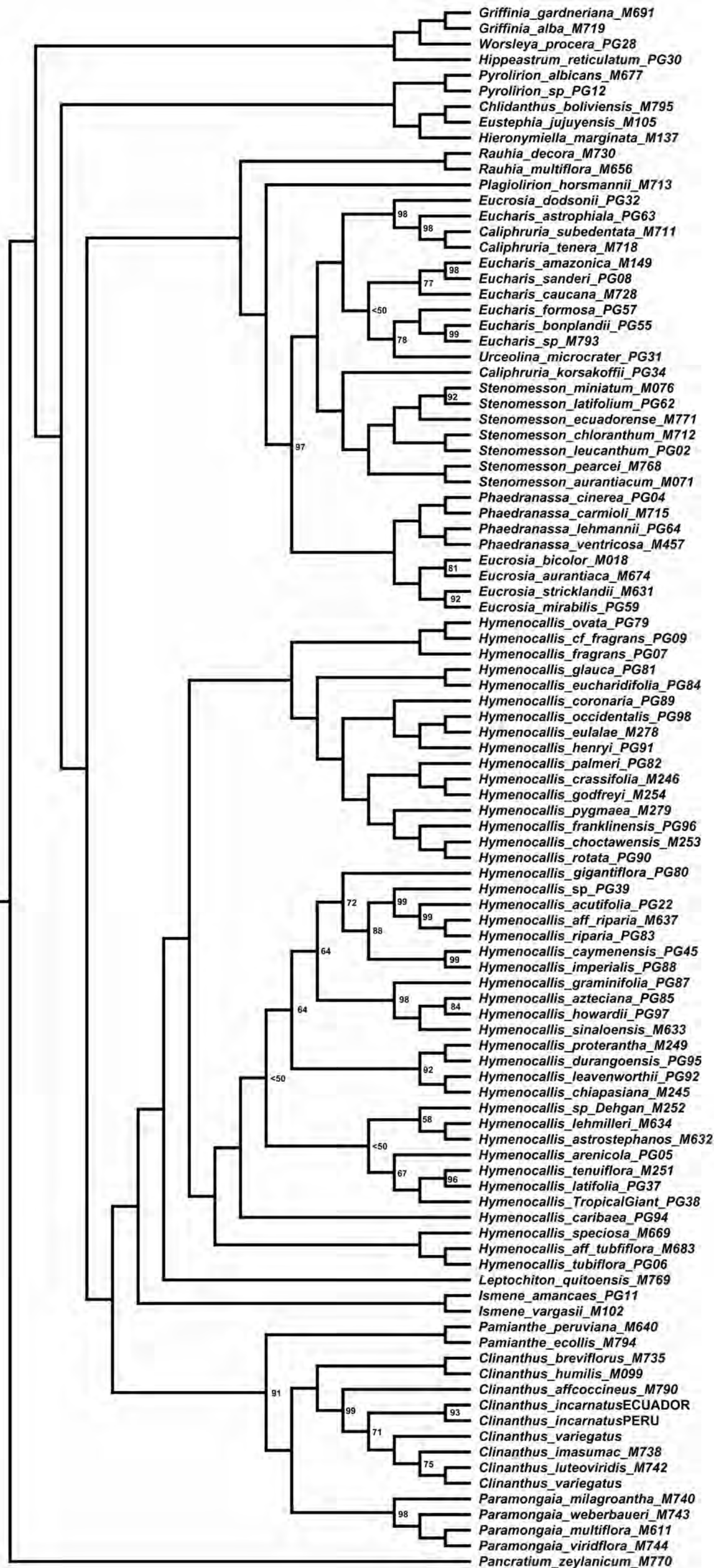

Supplement: Supplementary Figure 3 — Best tree from maximum likelihood analysis of partitioned 260 nuclear gene supermatrix (only genes amplified across 70% of the Andean tetraploid clade of Amaryllidaceae subfam. Amaryllidoideae), with bootstrap percentages < 100 shown above branches (all unmarked branches have 100% BP). Refer to Supplementary Table 1 for corrected taxon names. [file Data_Sheet_3.PDF]

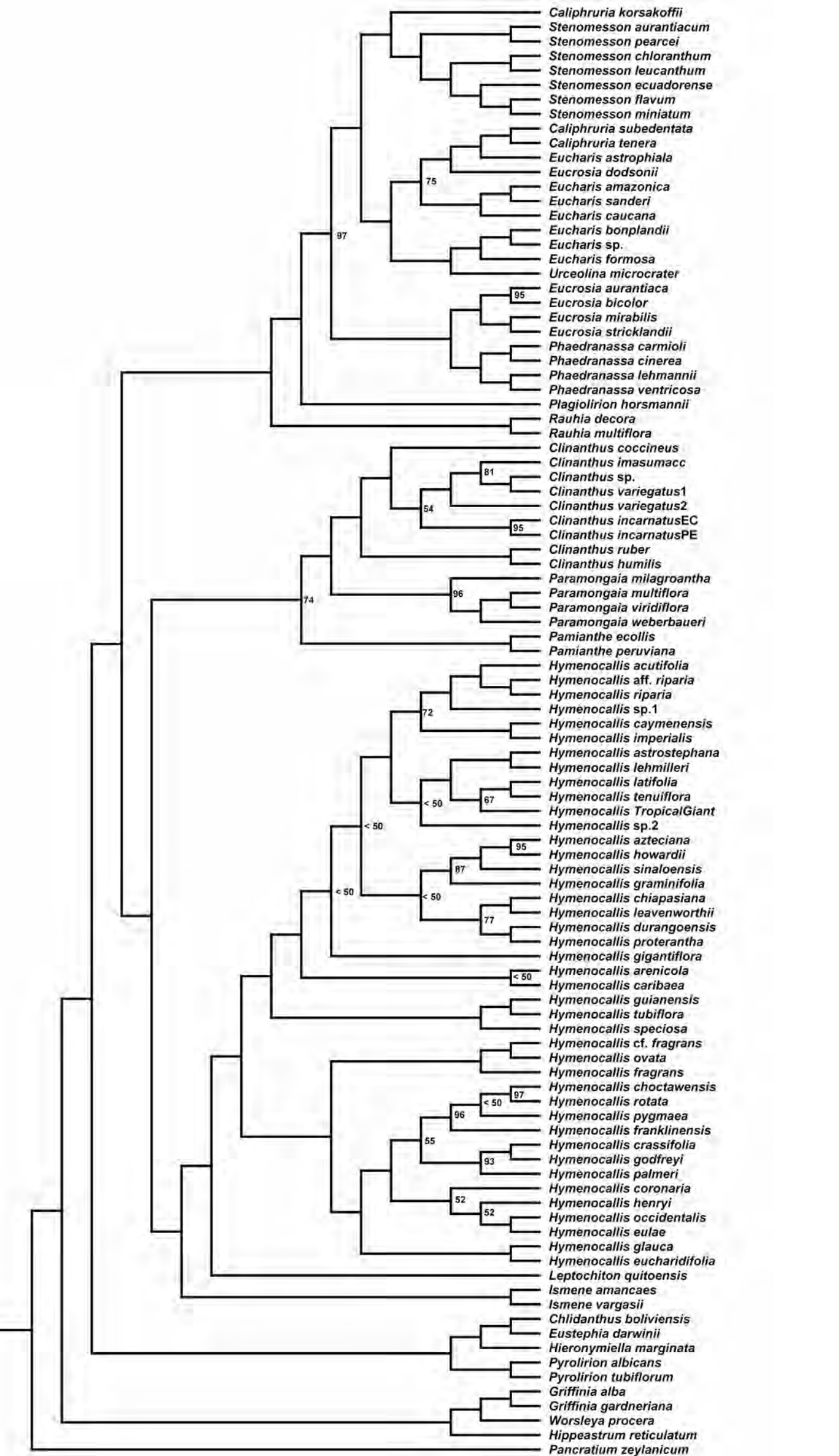

Supplement: Supplementary Figure 4 — Best tree from maximum likelihood analysis of partitioned 137 nuclear gene supermatrix (only genes amplified across 90% of the Andean tetraploid clade of Amaryllidaceae subfam. Amaryllidoideae), with bootstrap percentages < 100 shown above branches (all unmarked branches have 100% BP). [file Data_Sheet_4.PDF]

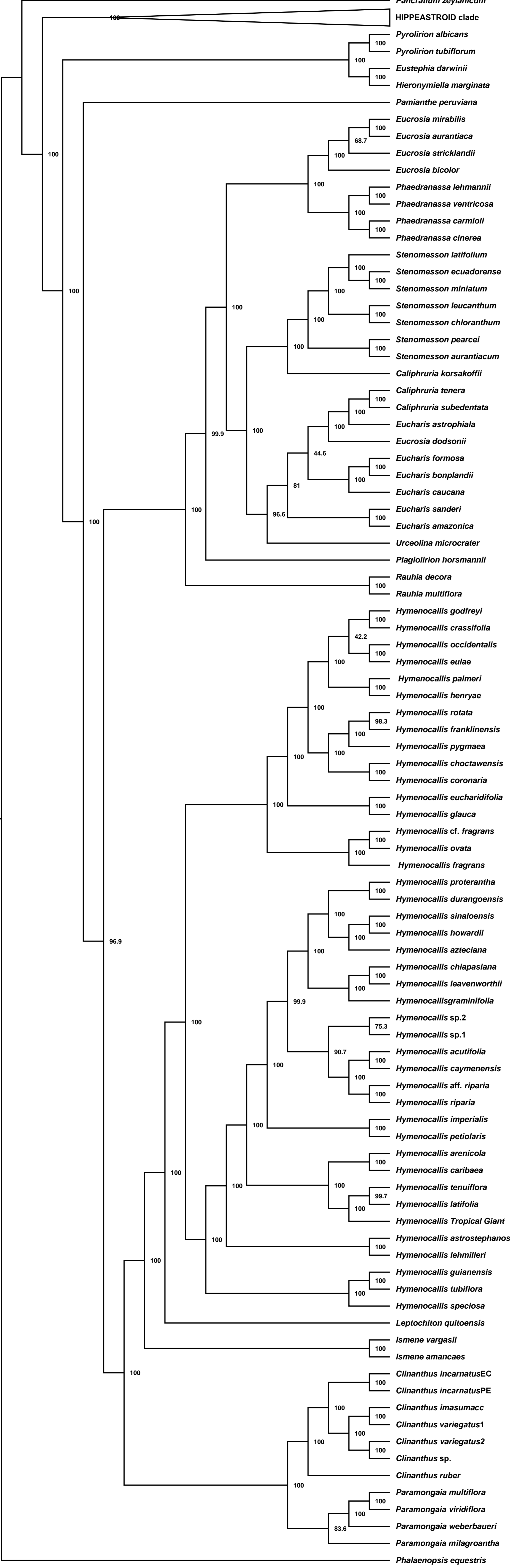

Supplement: Supplementary Figure 5 — Maximum likelihood tree with bootstrap using an earlier complete supermatrix of the Andean tetraploid clade of Amaryllidaceae in which consensus sequences of all paralogs with ambiguity codes were used. [file Data_Sheet_5.pdf]

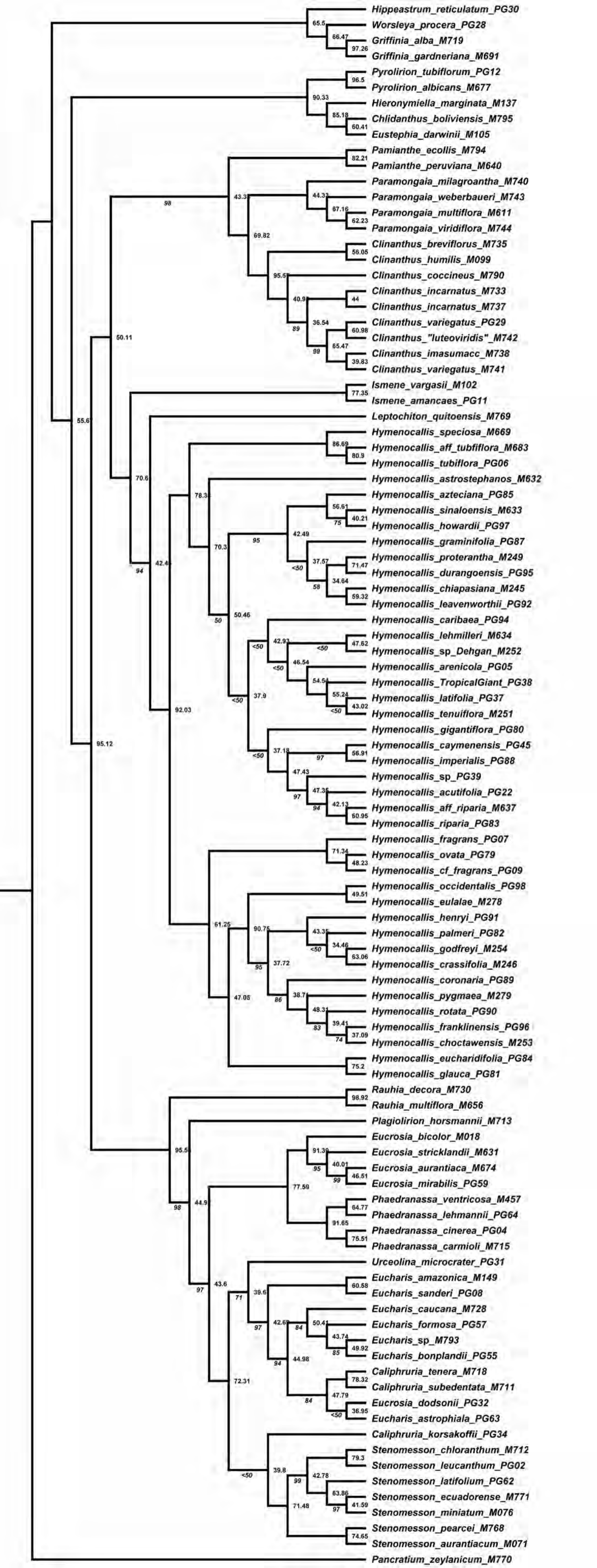

Supplement: Supplementary Figure 6 — Local posterior probability (LPP) coalescent species tree from ASTRAL III analysis of gene trees from 260 nuclear genes with 70% taxon coverage. LPP scores appear above the branches; bootstrap percentages < 100 shown below branches (all unmarked branches have 100% BP). Refer to Supplementary Table 1 for corrected taxon names. [file Data_Sheet_6.PDF]

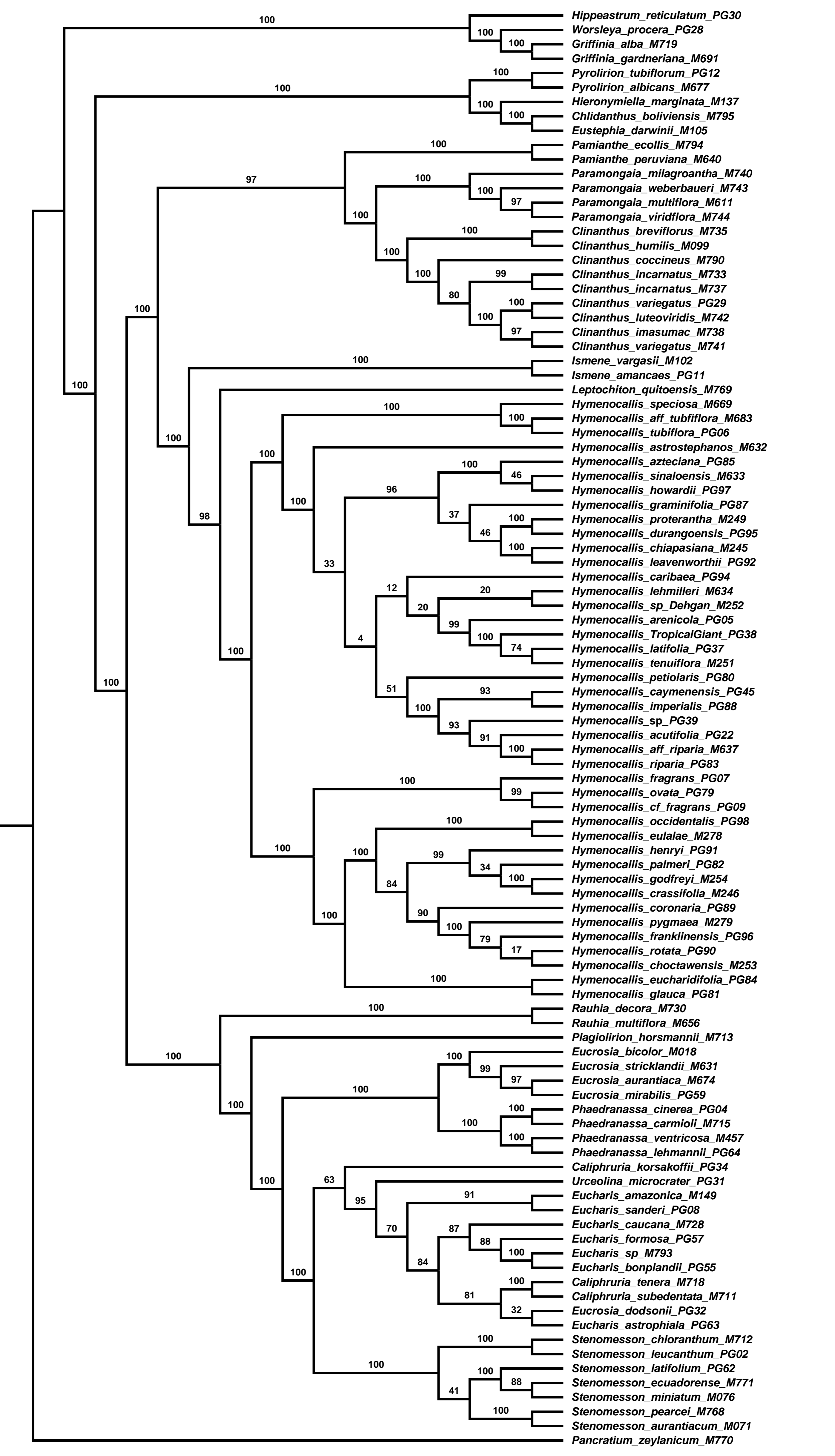

Supplement: Supplementary Figure 7 — Local posterior probability (LPP) coalescent species tree from ASTRAL III analysis of gene trees from 70 nuclear genes with 90% taxon coverage. LPP scores appear above the branches; bootstrap percentages < 100 shown below branches (all unmarked branches have 100% BP). Refer to Supplementary Table 1 for corrected taxon names. [file Data_Sheet_7.PDF]

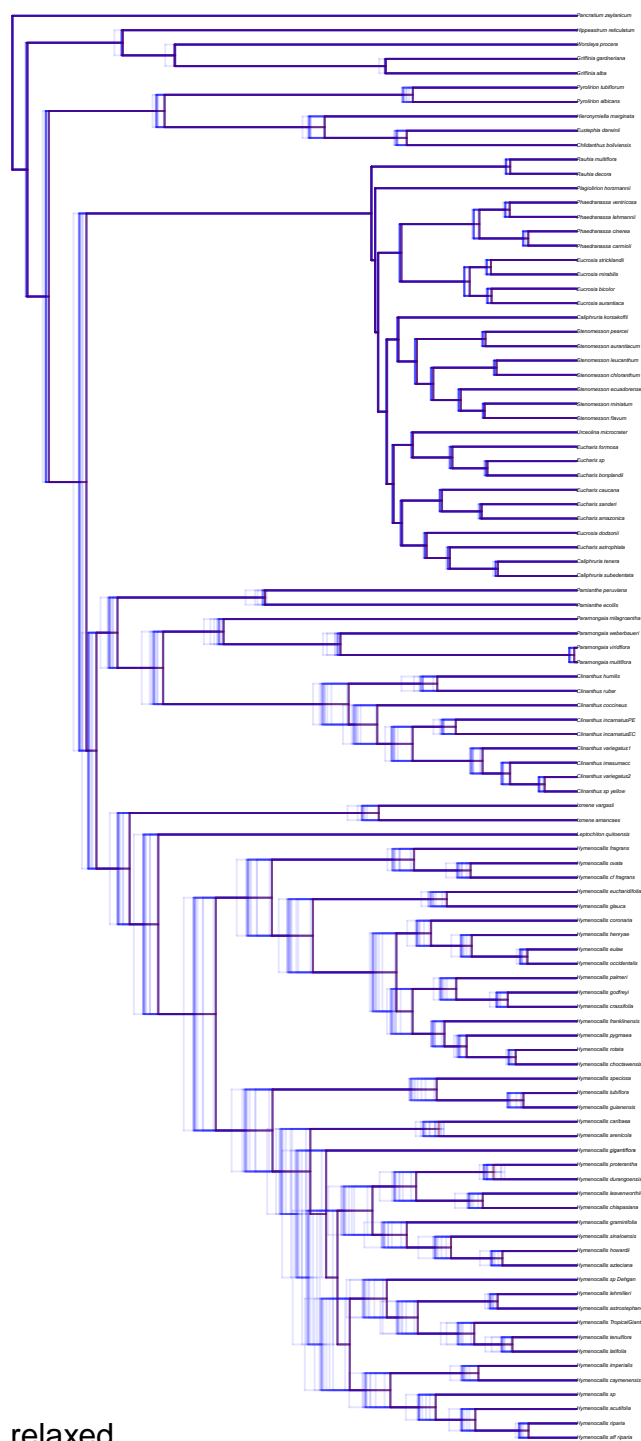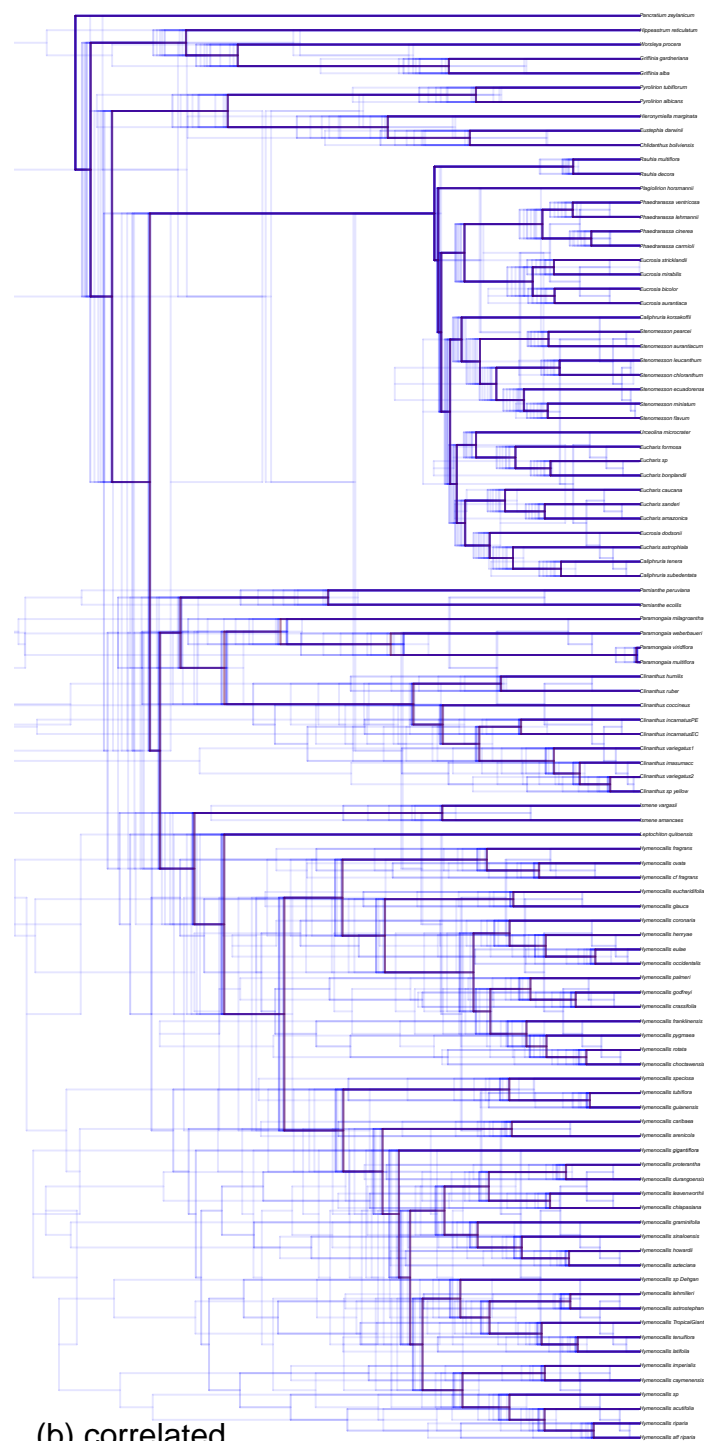

Supplement: Supplementary Figure 8 — Results of the densiTree comparison of relaxed and correlated penalized likelihood age-calibration of the best maximum likelihood tree found by RAxML using the 90% taxon coverage supermatrix and a range of values for λ (0–5). [file Data_Sheet_8.PDF]
