# Supplementary material for: Phylogenomics of the Andean Tetraploid Clade of the American Amaryllidaceae (Subfamily Amaryllidoideae): Unlocking a Polyploid Generic Radiation Abetted by Continental Geodynamics
Source: Front Plant Sci. 2020 Nov 5;11:582422. doi: 10.3389/fpls.2020.582422 (PMC7674842; doi:10.3389/fpls.2020.582422)
Supplement: Supplementary Table 3 — Event tally for DIVALIKE+J biogeographic analysis of the best 90% taxon coverage supermatrix tree found by maximum likelihood. Node numbers refer to Figure 10. [file Table_3.DOCX]

**Table S3.** Event tally for DIVALIKE+J biogeographic analysis of the best 90% taxon coverage supermatrix tree found by maximum likelihood. Node numbers refer to **Figure 10**.

NODE96:

EVENT MATRIX:

Dispersal:0

Vicariance:0

Extinction:0

Event Route:

C->C^C->C|C

PROBABILITY:

1.0000

NODE97:

EVENT MATRIX:

Dispersal:0

Vicariance:0

Extinction:0

Event Route:

G->G^G->G|G

PROBABILITY:

1.0000

NODE98:

EVENT MATRIX:

Dispersal:0

Vicariance:1

Extinction:0

Event Route:

CD->D|C

PROBABILITY:

0.9548

NODE99:

EVENT MATRIX:

Dispersal:0

Vicariance:1

Extinction:0

Event Route:

CDI->I|CD

PROBABILITY:

0.4909

NODE100:

EVENT MATRIX:

Dispersal:2

Vicariance:1

Extinction:0

Event Route:

CG->CDGI->CDI|G

PROBABILITY:

0.2110

NODE101:

EVENT MATRIX:

Dispersal:1

Vicariance:0

Extinction:0

Event Route:

C->C^C->CG^C->CG|C

PROBABILITY:

0.3385

NODE102:

EVENT MATRIX:

Dispersal:0

Vicariance:1

Extinction:0

Event Route:

AC->A|C

PROBABILITY:

0.6748

NODE103:

EVENT MATRIX:

Dispersal:0

Vicariance:0

Extinction:0

Event Route:

B->B^B->B|B

PROBABILITY:

1.0000

NODE104:

EVENT MATRIX:

Dispersal:0

Vicariance:1

Extinction:0

Event Route:

BI->I|B

PROBABILITY:

0.9071

NODE105:

EVENT MATRIX:

Dispersal:1

Vicariance:0

Extinction:0

Event Route:

BI->BI^I->I|BI

PROBABILITY:

0.4367

NODE106:

EVENT MATRIX:

Dispersal:0

Vicariance:1

Extinction:0

Event Route:

JK->J|K

PROBABILITY:

0.9545

NODE107:

EVENT MATRIX:

Dispersal:1

Vicariance:1

Extinction:0

Event Route:

BJ->BJK->B|JK

PROBABILITY:

0.3273

NODE108:

EVENT MATRIX:

Dispersal:2

Vicariance:0

Extinction:0

Event Route:

B->B^B->BIJ^B->BJ|BI

PROBABILITY:

0.0919

NODE109:

EVENT MATRIX:

Dispersal:0

Vicariance:0

Extinction:0

Event Route:

B->B^B->B|B

PROBABILITY:

1.0000

NODE110:

EVENT MATRIX:

Dispersal:0

Vicariance:1

Extinction:0

Event Route:

AB->A|B

PROBABILITY:

0.9578

NODE111:

EVENT MATRIX:

Dispersal:1

Vicariance:1

Extinction:0

Event Route:

BJ->ABJ->J|AB

PROBABILITY:

0.6193

NODE112:

EVENT MATRIX:

Dispersal:1

Vicariance:0

Extinction:0

Event Route:

B->B^B->BJ^B->BJ|B

PROBABILITY:

0.3327

NODE113:

EVENT MATRIX:

Dispersal:0

Vicariance:1

Extinction:0

Event Route:

ABC->B|AC

PROBABILITY:

0.2329

NODE114:

EVENT MATRIX:

Dispersal:0

Vicariance:0

Extinction:0

Event Route:

G->G^G->G|G

PROBABILITY:

1.0000

NODE115:

EVENT MATRIX:

Dispersal:0

Vicariance:0

Extinction:0

Event Route:

G->G^G->G|G

PROBABILITY:

1.0000

NODE116:

EVENT MATRIX:

Dispersal:0

Vicariance:0

Extinction:0

Event Route:

G->G^G->G|G

PROBABILITY:

1.0000

NODE117:

EVENT MATRIX:

Dispersal:0

Vicariance:1

Extinction:0

Event Route:

GM->M|G

PROBABILITY:

0.9663

NODE118:

EVENT MATRIX:

Dispersal:0

Vicariance:0

Extinction:0

Event Route:

B->B^B->B|B

PROBABILITY:

1.0000

NODE119:

EVENT MATRIX:

Dispersal:0

Vicariance:1

Extinction:0

Event Route:

BGM->B|GM

PROBABILITY:

0.5591

NODE120:

EVENT MATRIX:

Dispersal:2

Vicariance:0

Extinction:0

Event Route:

BG->BG^G->BGM^G->BGM|G

PROBABILITY:

0.2882

NODE121:

EVENT MATRIX:

Dispersal:3

Vicariance:0

Extinction:0

Event Route:

B->B^B->ABCG^B->BG|ABC

PROBABILITY:

0.1035

NODE122:

EVENT MATRIX:

Dispersal:0

Vicariance:0

Extinction:0

Event Route:

B->B^B->B|B

PROBABILITY:

0.6537

NODE123:

EVENT MATRIX:

Dispersal:0

Vicariance:0

Extinction:0

Event Route:

B->B^B->B|B

PROBABILITY:

1.0000

NODE124:

EVENT MATRIX:

Dispersal:0

Vicariance:0

Extinction:0

Event Route:

B->B^B->B|B

PROBABILITY:

0.9666

NODE125:

EVENT MATRIX:

Dispersal:0

Vicariance:0

Extinction:0

Event Route:

C->C^C->C|C

PROBABILITY:

1.0000

NODE126:

EVENT MATRIX:

Dispersal:0

Vicariance:1

Extinction:0

Event Route:

CF->F|C

PROBABILITY:

0.8311

NODE127:

EVENT MATRIX:

Dispersal:1

Vicariance:0

Extinction:0

Event Route:

C->C^C->CF^C->C|CF

PROBABILITY:

0.8077

NODE128:

EVENT MATRIX:

Dispersal:0

Vicariance:0

Extinction:0

Event Route:

G->G^G->G|G

PROBABILITY:

1.0000

NODE129:

EVENT MATRIX:

Dispersal:0

Vicariance:1

Extinction:0

Event Route:

CG->G|C

PROBABILITY:

0.8733

NODE130:

EVENT MATRIX:

Dispersal:1

Vicariance:1

Extinction:0

Event Route:

DG->CDG->D|CG

PROBABILITY:

0.3268

NODE131:

EVENT MATRIX:

Dispersal:0

Vicariance:1

Extinction:0

Event Route:

CE->C|E

PROBABILITY:

0.9250

NODE132:

EVENT MATRIX:

Dispersal:1

Vicariance:1

Extinction:0

Event Route:

CDE->CDEG->CE|DG

PROBABILITY:

0.0956

NODE133:

EVENT MATRIX:

Dispersal:0

Vicariance:0

Extinction:0

Event Route:

F->F^F->F|F

PROBABILITY:

1.0000

NODE134:

EVENT MATRIX:

Dispersal:0

Vicariance:1

Extinction:0

Event Route:

DF->D|F

PROBABILITY:

0.9680

NODE135:

EVENT MATRIX:

Dispersal:1

Vicariance:1

Extinction:0

Event Route:

DG->DFG->G|DF

PROBABILITY:

0.4363

NODE136:

EVENT MATRIX:

Dispersal:3

Vicariance:0

Extinction:0

Event Route:

D->D^D->CDEG^D->DG|CDE

PROBABILITY:

0.0621

NODE137:

EVENT MATRIX:

Dispersal:0

Vicariance:1

Extinction:0

Event Route:

BF->B|F

PROBABILITY:

0.9642

NODE138:

EVENT MATRIX:

Dispersal:1

Vicariance:1

Extinction:0

Event Route:

BD->BDF->BF|D

PROBABILITY:

0.1060

NODE139:

EVENT MATRIX:

Dispersal:0

Vicariance:0

Extinction:0

Event Route:

M->M^M->M|M

PROBABILITY:

1.0000

NODE140:

EVENT MATRIX:

Dispersal:0

Vicariance:0

Extinction:0

Event Route:

M->M^M->M|M

PROBABILITY:

1.0000

NODE141:

EVENT MATRIX:

Dispersal:0

Vicariance:0

Extinction:0

Event Route:

M->M^M->M|M

PROBABILITY:

1.0000

NODE142:

EVENT MATRIX:

Dispersal:0

Vicariance:1

Extinction:0

Event Route:

MO->O|M

PROBABILITY:

0.9052

NODE143:

EVENT MATRIX:

Dispersal:1

Vicariance:0

Extinction:0

Event Route:

M->M^M->MO^M->MO|M

PROBABILITY:

0.9030

NODE144:

EVENT MATRIX:

Dispersal:0

Vicariance:0

Extinction:0

Event Route:

M->M^M->M|M

PROBABILITY:

1.0000

NODE145:

EVENT MATRIX:

Dispersal:0

Vicariance:1

Extinction:0

Event Route:

MO->O|M

PROBABILITY:

0.8709

NODE146:

EVENT MATRIX:

Dispersal:1

Vicariance:0

Extinction:0

Event Route:

MO->MO^O->O|MO

PROBABILITY:

0.4656

NODE147:

EVENT MATRIX:

Dispersal:1

Vicariance:0

Extinction:0

Event Route:

M->M^M->MO^M->MO|M

PROBABILITY:

0.3412

NODE148:

EVENT MATRIX:

Dispersal:0

Vicariance:0

Extinction:0

Event Route:

M->M^M->M|M

PROBABILITY:

0.6374

NODE149:

EVENT MATRIX:

Dispersal:0

Vicariance:0

Extinction:0

Event Route:

M->M^M->M|M

PROBABILITY:

0.9962

NODE150:

EVENT MATRIX:

Dispersal:0

Vicariance:0

Extinction:0

Event Route:

M->M^M->M|M

PROBABILITY:

1.0000

NODE151:

EVENT MATRIX:

Dispersal:0

Vicariance:0

Extinction:0

Event Route:

M->M^M->M|M

PROBABILITY:

1.0000

NODE152:

EVENT MATRIX:

Dispersal:0

Vicariance:0

Extinction:0

Event Route:

M->M^M->M|M

PROBABILITY:

1.0000

NODE153:

EVENT MATRIX:

Dispersal:0

Vicariance:0

Extinction:0

Event Route:

M->M^M->M|M

PROBABILITY:

1.0000

NODE154:

EVENT MATRIX:

Dispersal:0

Vicariance:0

Extinction:0

Event Route:

M->M^M->M|M

PROBABILITY:

1.0000

NODE155:

EVENT MATRIX:

Dispersal:0

Vicariance:0

Extinction:0

Event Route:

M->M^M->M|M

PROBABILITY:

1.0000

NODE156:

EVENT MATRIX:

Dispersal:0

Vicariance:0

Extinction:0

Event Route:

M->M^M->M|M

PROBABILITY:

1.0000

NODE157:

EVENT MATRIX:

Dispersal:0

Vicariance:0

Extinction:0

Event Route:

M->M^M->M|M

PROBABILITY:

1.0000

NODE158:

EVENT MATRIX:

Dispersal:0

Vicariance:0

Extinction:0

Event Route:

M->M^M->M|M

PROBABILITY:

1.0000

NODE159:

EVENT MATRIX:

Dispersal:0

Vicariance:0

Extinction:0

Event Route:

O->O^O->O|O

PROBABILITY:

1.0000

NODE160:

EVENT MATRIX:

Dispersal:0

Vicariance:1

Extinction:0

Event Route:

MO->O|M

PROBABILITY:

0.9277

NODE161:

EVENT MATRIX:

Dispersal:0

Vicariance:0

Extinction:0

Event Route:

N->N^N->N|N

PROBABILITY:

1.0000

NODE162:

EVENT MATRIX:

Dispersal:0

Vicariance:1

Extinction:0

Event Route:

NO->O|N

PROBABILITY:

0.9499

NODE163:

EVENT MATRIX:

Dispersal:2

Vicariance:0

Extinction:0

Event Route:

O->O^O->MNO^O->NO|MO

PROBABILITY:

0.8308

NODE164:

EVENT MATRIX:

Dispersal:0

Vicariance:0

Extinction:0

Event Route:

O->O^O->O|O

PROBABILITY:

1.0000

NODE165:

EVENT MATRIX:

Dispersal:0

Vicariance:0

Extinction:0

Event Route:

O->O^O->O|O

PROBABILITY:

1.0000

NODE166:

EVENT MATRIX:

Dispersal:0

Vicariance:0

Extinction:0

Event Route:

H->H^H->H|H

PROBABILITY:

1.0000

NODE167:

EVENT MATRIX:

Dispersal:0

Vicariance:0

Extinction:0

Event Route:

H->H^H->H|H

PROBABILITY:

1.0000

NODE168:

EVENT MATRIX:

Dispersal:0

Vicariance:0

Extinction:0

Event Route:

H->H^H->H|H

PROBABILITY:

1.0000

NODE169:

EVENT MATRIX:

Dispersal:0

Vicariance:0

Extinction:0

Event Route:

H->H^H->H|H

PROBABILITY:

1.0000

NODE170:

EVENT MATRIX:

Dispersal:0

Vicariance:0

Extinction:0

Event Route:

H->H^H->H|H

PROBABILITY:

1.0000

NODE171:

EVENT MATRIX:

Dispersal:0

Vicariance:0

Extinction:0

Event Route:

H->H^H->H|H

PROBABILITY:

1.0000

NODE172:

EVENT MATRIX:

Dispersal:0

Vicariance:0

Extinction:0

Event Route:

H->H^H->H|H

PROBABILITY:

1.0000

NODE173:

EVENT MATRIX:

Dispersal:0

Vicariance:0

Extinction:0

Event Route:

H->H^H->H|H

PROBABILITY:

1.0000

NODE174:

EVENT MATRIX:

Dispersal:0

Vicariance:0

Extinction:0

Event Route:

H->H^H->H|H

PROBABILITY:

1.0000

NODE175:

EVENT MATRIX:

Dispersal:0

Vicariance:0

Extinction:0

Event Route:

H->H^H->H|H

PROBABILITY:

1.0000

NODE176:

EVENT MATRIX:

Dispersal:0

Vicariance:0

Extinction:0

Event Route:

M->M^M->M|M

PROBABILITY:

1.0000

NODE177:

EVENT MATRIX:

Dispersal:0

Vicariance:1

Extinction:0

Event Route:

HM->M|H

PROBABILITY:

0.9455

NODE178:

EVENT MATRIX:

Dispersal:0

Vicariance:1

Extinction:0

Event Route:

HMO->HM|O

PROBABILITY:

0.3234

NODE179:

EVENT MATRIX:

Dispersal:2

Vicariance:0

Extinction:0

Event Route:

O->O^O->HMO^O->HMO|O

PROBABILITY:

0.2944

NODE180:

EVENT MATRIX:

Dispersal:0

Vicariance:1

Extinction:0

Event Route:

DO->D|O

PROBABILITY:

0.8065

NODE181:

EVENT MATRIX:

Dispersal:0

Vicariance:1

Extinction:0

Event Route:

CD->D|C

PROBABILITY:

0.9696

NODE182:

EVENT MATRIX:

Dispersal:2

Vicariance:0

Extinction:0

Event Route:

D->D^D->CDO^D->CD|DO

PROBABILITY:

0.7938

NODE183:

EVENT MATRIX:

Dispersal:1

Vicariance:0

Extinction:0

Event Route:

D->D^D->BD^D->D|BD

PROBABILITY:

0.1040

NODE184:

EVENT MATRIX:

Dispersal:0

Vicariance:1

Extinction:0

Event Route:

BD->D|B

PROBABILITY:

0.2534

NODE185:

EVENT MATRIX:

Dispersal:0

Vicariance:0

Extinction:0

Event Route:

C->C^C->C|C

PROBABILITY:

1.0000

NODE186:

EVENT MATRIX:

Dispersal:0

Vicariance:1

Extinction:0

Event Route:

CL->L|C

PROBABILITY:

0.9847

NODE187:

EVENT MATRIX:

Dispersal:2

Vicariance:0

Extinction:0

Event Route:

D->D^D->BDI^D->D|BDI

PROBABILITY:

0.7468

NODE188:

EVENT MATRIX:

Dispersal:0

Vicariance:1

Extinction:0

Event Route:

CDL->D|CL

PROBABILITY:

0.3340

NODE189:

EVENT MATRIX:

Dispersal:2

Vicariance:0

Extinction:0

Event Route:

BCD->BCD^D->BCDL^D->CDL|BD

PROBABILITY:

0.0565

===================

Dispersal Between Areas:

B->A:1.5

B->C:1

B->F:0.5

B->G:1

B->I:1

B->J:2

B->K:0.5

B->L:0.3333333

B->M:0.5

C->D:0.5

C->F:1

C->G:1.333333

C->I:0.5

C->L:0.3333333

D->B:2

D->C:2.5

D->E:1

D->F:1

D->G:1.333333

D->I:1

D->L:0.3333333

D->O:1

E->G:0.3333333

G->C:0.5

G->D:0.5

G->F:0.5

G->I:0.5

G->M:0.5

J->A:0.5

J->K:0.5

M->O:2

O->H:1

O->M:2

O->N:1

Speciation Within Areas:

B:9

C:5

D:5

F:1

G:6

H:10

I:1

M:18

N:1

O:6

Dispersal Table:

from to within

A 0.00 2.00 0

B 8.33 2.00 9

C 3.67 4.00 5

D 10.17 1.00 5

E 0.33 1.00 0

F 0.00 3.00 1

G 2.50 4.00 6

H 0.00 1.00 10

I 0.00 3.00 1

J 1.00 2.00 0

K 0.00 1.00 0

L 0.00 1.00 0

M 2.00 3.00 18

N 0.00 1.00 1

O 4.00 3.00 6

===================

Global Cost:

Global Dispersal: 36

Global Vicariance: 32

Global Extinction: 0
