# Supplementary material for: Phylogenomics of the Andean Tetraploid Clade of the American Amaryllidaceae (Subfamily Amaryllidoideae): Unlocking a Polyploid Generic Radiation Abetted by Continental Geodynamics
Source: Front Plant Sci. 2020 Nov 5;11:582422. doi: 10.3389/fpls.2020.582422 (PMC7674842; doi:10.3389/fpls.2020.582422)
Supplement: Supplementary Table 4 — Event tally for BAYAREALIKE +J biogeographic analysis of the partial plastome supermatrix best ML tree. Node numbers refer to Supplementary Figure 10. [file Table_4.DOCX]

**Supplemental Table S4.** Event tally for BAYAREALIKE+J biogeographic analysis of the partial plastome supermatrix best maximum likelihood tree. Node numbers refer to **Supplementary Figure S9**.

NODE96:

EVENT MATRIX:

Dispersal:2

Vicariance:1

Extinction:0

Event Route:

A->AJ->A|J

PROBABILITY:

0.7462

NODE97:

EVENT MATRIX:

Dispersal:0

Vicariance:0

Extinction:0

Event Route:

B->B^B->B|B

PROBABILITY:

1.0000

NODE98:

EVENT MATRIX:

Dispersal:0

Vicariance:0

Extinction:0

Event Route:

B->B^B->B|B

PROBABILITY:

1.0000

NODE99:

EVENT MATRIX:

Dispersal:2

Vicariance:1

Extinction:0

Event Route:

A->AB->A|B

PROBABILITY:

0.5022

NODE100:

EVENT MATRIX:

Dispersal:0

Vicariance:0

Extinction:0

Event Route:

A->A^A->A|A

PROBABILITY:

0.1882

NODE101:

EVENT MATRIX:

Dispersal:2

Vicariance:1

Extinction:0

Event Route:

C->CI->C|I

PROBABILITY:

0.9952

NODE102:

EVENT MATRIX:

Dispersal:0

Vicariance:0

Extinction:0

Event Route:

C->C^C->C|C

PROBABILITY:

0.9951

NODE103:

EVENT MATRIX:

Dispersal:0

Vicariance:0

Extinction:0

Event Route:

C->C^C->C|C

PROBABILITY:

0.9999

NODE104:

EVENT MATRIX:

Dispersal:0

Vicariance:0

Extinction:0

Event Route:

G->G^G->G|G

PROBABILITY:

1.0000

NODE105:

EVENT MATRIX:

Dispersal:2

Vicariance:1

Extinction:0

Event Route:

D->DG->D|G

PROBABILITY:

0.4999

NODE106:

EVENT MATRIX:

Dispersal:2

Vicariance:1

Extinction:0

Event Route:

C->CD->D|C

PROBABILITY:

0.2496

NODE107:

EVENT MATRIX:

Dispersal:3

Vicariance:1

Extinction:1

Event Route:

B->->AC->C|A

PROBABILITY:

0.1230

NODE108:

EVENT MATRIX:

Dispersal:2

Vicariance:1

Extinction:0

Event Route:

B->BJ->B|J

PROBABILITY:

0.9951

NODE109:

EVENT MATRIX:

Dispersal:0

Vicariance:0

Extinction:0

Event Route:

B->B^B->B|B

PROBABILITY:

0.9950

NODE110:

EVENT MATRIX:

Dispersal:0

Vicariance:0

Extinction:0

Event Route:

B->B^B->B|B

PROBABILITY:

0.4869

NODE111:

EVENT MATRIX:

Dispersal:2

Vicariance:1

Extinction:0

Event Route:

B->BK->K|B

PROBABILITY:

0.9800

NODE112:

EVENT MATRIX:

Dispersal:0

Vicariance:0

Extinction:0

Event Route:

I->I^I->I|I

PROBABILITY:

0.9999

NODE113:

EVENT MATRIX:

Dispersal:0

Vicariance:0

Extinction:0

Event Route:

G->G^G->G|G

PROBABILITY:

1.0000

NODE114:

EVENT MATRIX:

Dispersal:0

Vicariance:0

Extinction:0

Event Route:

G->G^G->G|G

PROBABILITY:

1.0000

NODE115:

EVENT MATRIX:

Dispersal:0

Vicariance:0

Extinction:0

Event Route:

G->G^G->G|G

PROBABILITY:

1.0000

NODE116:

EVENT MATRIX:

Dispersal:2

Vicariance:1

Extinction:0

Event Route:

G->GM->M|G

PROBABILITY:

0.6658

NODE117:

EVENT MATRIX:

Dispersal:0

Vicariance:0

Extinction:0

Event Route:

B->B^B->B|B

PROBABILITY:

1.0000

NODE118:

EVENT MATRIX:

Dispersal:2

Vicariance:1

Extinction:0

Event Route:

B->BG->B|G

PROBABILITY:

0.4407

NODE119:

EVENT MATRIX:

Dispersal:2

Vicariance:1

Extinction:0

Event Route:

B->BG->B|G

PROBABILITY:

0.4360

NODE120:

EVENT MATRIX:

Dispersal:0

Vicariance:0

Extinction:0

Event Route:

B->B^B->B|B

PROBABILITY:

0.6545

NODE121:

EVENT MATRIX:

Dispersal:2

Vicariance:1

Extinction:0

Event Route:

B->BI->B|I

PROBABILITY:

0.9816

NODE122:

EVENT MATRIX:

Dispersal:0

Vicariance:0

Extinction:0

Event Route:

B->B^B->B|B

PROBABILITY:

0.9700

NODE123:

EVENT MATRIX:

Dispersal:0

Vicariance:0

Extinction:0

Event Route:

B->B^B->B|B

PROBABILITY:

0.9999

NODE124:

EVENT MATRIX:

Dispersal:0

Vicariance:0

Extinction:0

Event Route:

B->B^B->B|B

PROBABILITY:

0.9805

NODE125:

EVENT MATRIX:

Dispersal:2

Vicariance:1

Extinction:0

Event Route:

B->BF->B|F

PROBABILITY:

0.9217

NODE126:

EVENT MATRIX:

Dispersal:0

Vicariance:0

Extinction:0

Event Route:

B->B^B->B|B

PROBABILITY:

0.8073

NODE127:

EVENT MATRIX:

Dispersal:2

Vicariance:1

Extinction:0

Event Route:

C->CE->E|C

PROBABILITY:

0.9670

NODE128:

EVENT MATRIX:

Dispersal:2

Vicariance:1

Extinction:0

Event Route:

C->CD->D|C

PROBABILITY:

0.9143

NODE129:

EVENT MATRIX:

Dispersal:2

Vicariance:1

Extinction:0

Event Route:

C->CG->G|C

PROBABILITY:

0.9777

NODE130:

EVENT MATRIX:

Dispersal:0

Vicariance:0

Extinction:0

Event Route:

C->C^C->C|C

PROBABILITY:

0.9605

NODE131:

EVENT MATRIX:

Dispersal:2

Vicariance:1

Extinction:0

Event Route:

C->CF->F|C

PROBABILITY:

0.9392

NODE132:

EVENT MATRIX:

Dispersal:2

Vicariance:1

Extinction:0

Event Route:

C->CG->G|C

PROBABILITY:

0.8972

NODE133:

EVENT MATRIX:

Dispersal:0

Vicariance:0

Extinction:0

Event Route:

C->C^C->C|C

PROBABILITY:

0.8448

NODE134:

EVENT MATRIX:

Dispersal:0

Vicariance:0

Extinction:0

Event Route:

C->C^C->C|C

PROBABILITY:

0.9070

NODE135:

EVENT MATRIX:

Dispersal:2

Vicariance:1

Extinction:0

Event Route:

G->CG->G|C

PROBABILITY:

0.4780

NODE136:

EVENT MATRIX:

Dispersal:0

Vicariance:0

Extinction:0

Event Route:

F->F^F->F|F

PROBABILITY:

1.0000

NODE137:

EVENT MATRIX:

Dispersal:2

Vicariance:1

Extinction:0

Event Route:

D->DF->D|F

PROBABILITY:

0.9511

NODE138:

EVENT MATRIX:

Dispersal:2

Vicariance:1

Extinction:0

Event Route:

D->DG->D|G

PROBABILITY:

0.4323

NODE139:

EVENT MATRIX:

Dispersal:0

Vicariance:0

Extinction:0

Event Route:

M->M^M->M|M

PROBABILITY:

1.0000

NODE140:

EVENT MATRIX:

Dispersal:0

Vicariance:0

Extinction:0

Event Route:

M->M^M->M|M

PROBABILITY:

1.0000

NODE141:

EVENT MATRIX:

Dispersal:2

Vicariance:1

Extinction:0

Event Route:

M->MO->O|M

PROBABILITY:

0.9882

NODE142:

EVENT MATRIX:

Dispersal:0

Vicariance:0

Extinction:0

Event Route:

M->M^M->M|M

PROBABILITY:

0.9812

NODE143:

EVENT MATRIX:

Dispersal:0

Vicariance:0

Extinction:0

Event Route:

H->H^H->H|H

PROBABILITY:

1.0000

NODE144:

EVENT MATRIX:

Dispersal:0

Vicariance:0

Extinction:0

Event Route:

H->H^H->H|H

PROBABILITY:

1.0000

NODE145:

EVENT MATRIX:

Dispersal:0

Vicariance:0

Extinction:0

Event Route:

H->H^H->H|H

PROBABILITY:

1.0000

NODE146:

EVENT MATRIX:

Dispersal:0

Vicariance:0

Extinction:0

Event Route:

H->H^H->H|H

PROBABILITY:

1.0000

NODE147:

EVENT MATRIX:

Dispersal:0

Vicariance:0

Extinction:0

Event Route:

H->H^H->H|H

PROBABILITY:

1.0000

NODE148:

EVENT MATRIX:

Dispersal:0

Vicariance:0

Extinction:0

Event Route:

H->H^H->H|H

PROBABILITY:

1.0000

NODE149:

EVENT MATRIX:

Dispersal:0

Vicariance:0

Extinction:0

Event Route:

H->H^H->H|H

PROBABILITY:

1.0000

NODE150:

EVENT MATRIX:

Dispersal:0

Vicariance:0

Extinction:0

Event Route:

H->H^H->H|H

PROBABILITY:

1.0000

NODE151:

EVENT MATRIX:

Dispersal:0

Vicariance:0

Extinction:0

Event Route:

H->H^H->H|H

PROBABILITY:

1.0000

NODE152:

EVENT MATRIX:

Dispersal:0

Vicariance:0

Extinction:0

Event Route:

H->H^H->H|H

PROBABILITY:

1.0000

NODE153:

EVENT MATRIX:

Dispersal:2

Vicariance:1

Extinction:0

Event Route:

M->HM->H|M

PROBABILITY:

0.9742

NODE154:

EVENT MATRIX:

Dispersal:0

Vicariance:0

Extinction:0

Event Route:

O->O^O->O|O

PROBABILITY:

1.0000

NODE155:

EVENT MATRIX:

Dispersal:0

Vicariance:0

Extinction:0

Event Route:

O->O^O->O|O

PROBABILITY:

1.0000

NODE156:

EVENT MATRIX:

Dispersal:2

Vicariance:1

Extinction:0

Event Route:

M->MO->O|M

PROBABILITY:

0.9556

NODE157:

EVENT MATRIX:

Dispersal:0

Vicariance:0

Extinction:0

Event Route:

M->M^M->M|M

PROBABILITY:

1.0000

NODE158:

EVENT MATRIX:

Dispersal:0

Vicariance:0

Extinction:0

Event Route:

M->M^M->M|M

PROBABILITY:

0.9670

NODE159:

EVENT MATRIX:

Dispersal:0

Vicariance:0

Extinction:0

Event Route:

O->O^O->O|O

PROBABILITY:

1.0000

NODE160:

EVENT MATRIX:

Dispersal:0

Vicariance:0

Extinction:0

Event Route:

O->O^O->O|O

PROBABILITY:

1.0000

NODE161:

EVENT MATRIX:

Dispersal:2

Vicariance:1

Extinction:0

Event Route:

M->MO->M|O

PROBABILITY:

0.9952

NODE162:

EVENT MATRIX:

Dispersal:0

Vicariance:0

Extinction:0

Event Route:

M->M^M->M|M

PROBABILITY:

1.0000

NODE163:

EVENT MATRIX:

Dispersal:0

Vicariance:0

Extinction:0

Event Route:

M->M^M->M|M

PROBABILITY:

0.9952

NODE164:

EVENT MATRIX:

Dispersal:0

Vicariance:0

Extinction:0

Event Route:

M->M^M->M|M

PROBABILITY:

1.0000

NODE165:

EVENT MATRIX:

Dispersal:0

Vicariance:0

Extinction:0

Event Route:

M->M^M->M|M

PROBABILITY:

1.0000

NODE166:

EVENT MATRIX:

Dispersal:0

Vicariance:0

Extinction:0

Event Route:

M->M^M->M|M

PROBABILITY:

1.0000

NODE167:

EVENT MATRIX:

Dispersal:0

Vicariance:0

Extinction:0

Event Route:

M->M^M->M|M

PROBABILITY:

1.0000

NODE168:

EVENT MATRIX:

Dispersal:0

Vicariance:0

Extinction:0

Event Route:

M->M^M->M|M

PROBABILITY:

1.0000

NODE169:

EVENT MATRIX:

Dispersal:0

Vicariance:0

Extinction:0

Event Route:

M->M^M->M|M

PROBABILITY:

1.0000

NODE170:

EVENT MATRIX:

Dispersal:0

Vicariance:0

Extinction:0

Event Route:

M->M^M->M|M

PROBABILITY:

1.0000

NODE171:

EVENT MATRIX:

Dispersal:0

Vicariance:0

Extinction:0

Event Route:

M->M^M->M|M

PROBABILITY:

1.0000

NODE172:

EVENT MATRIX:

Dispersal:0

Vicariance:0

Extinction:0

Event Route:

M->M^M->M|M

PROBABILITY:

1.0000

NODE173:

EVENT MATRIX:

Dispersal:0

Vicariance:0

Extinction:0

Event Route:

M->M^M->M|M

PROBABILITY:

1.0000

NODE174:

EVENT MATRIX:

Dispersal:2

Vicariance:1

Extinction:0

Event Route:

M->MO->O|M

PROBABILITY:

0.9905

NODE175:

EVENT MATRIX:

Dispersal:0

Vicariance:0

Extinction:0

Event Route:

M->M^M->M|M

PROBABILITY:

0.9858

NODE176:

EVENT MATRIX:

Dispersal:0

Vicariance:0

Extinction:0

Event Route:

M->M^M->M|M

PROBABILITY:

0.9799

NODE177:

EVENT MATRIX:

Dispersal:0

Vicariance:0

Extinction:0

Event Route:

N->N^N->N|N

PROBABILITY:

1.0000

NODE178:

EVENT MATRIX:

Dispersal:2

Vicariance:1

Extinction:0

Event Route:

O->NO->O|N

PROBABILITY:

0.5037

NODE179:

EVENT MATRIX:

Dispersal:2

Vicariance:1

Extinction:0

Event Route:

M->MO->O|M

PROBABILITY:

0.2477

NODE180:

EVENT MATRIX:

Dispersal:2

Vicariance:1

Extinction:0

Event Route:

D->DM->D|M

PROBABILITY:

0.4847

NODE181:

EVENT MATRIX:

Dispersal:2

Vicariance:1

Extinction:0

Event Route:

D->CD->D|C

PROBABILITY:

0.9808

NODE182:

EVENT MATRIX:

Dispersal:0

Vicariance:0

Extinction:0

Event Route:

D->D^D->D|D

PROBABILITY:

0.9388

NODE183:

EVENT MATRIX:

Dispersal:0

Vicariance:0

Extinction:0

Event Route:

D->D^D->D|D

PROBABILITY:

0.7932

NODE184:

EVENT MATRIX:

Dispersal:0

Vicariance:1

Extinction:0

Event Route:

BD->D|B

PROBABILITY:

0.5526

NODE185:

EVENT MATRIX:

Dispersal:0

Vicariance:0

Extinction:0

Event Route:

C->C^C->C|C

PROBABILITY:

1.0000

NODE186:

EVENT MATRIX:

Dispersal:2

Vicariance:1

Extinction:0

Event Route:

C->CL->L|C

PROBABILITY:

0.5000

NODE187:

EVENT MATRIX:

Dispersal:1

Vicariance:0

Extinction:0

Event Route:

BDI->BDI^D->D|BDI

PROBABILITY:

0.9938

NODE188:

EVENT MATRIX:

Dispersal:1

Vicariance:1

Extinction:0

Event Route:

BDI->BCDI->BDI|C

PROBABILITY:

0.4935

NODE189:

EVENT MATRIX:

Dispersal:2

Vicariance:0

Extinction:0

Event Route:

BDI->BDI^B^D->BDI|BD

PROBABILITY:

0.6858

===================

Dispersal Between Areas:

A->B:1

A->J:1

B->A:1

B->C:1.333333

B->F:1

B->G:2

B->I:1

B->J:1

B->K:1

C->D:2

C->E:1

C->F:1

C->G:2

C->I:1

C->L:1

D->C:1.333333

D->F:1

D->G:2

D->M:1

G->C:1

G->M:1

I->C:0.3333333

M->H:1

M->O:5

O->N:1

Speciation Within Areas:

A:1

B:11

C:6

D:4

F:1

G:4

H:10

I:1

M:19

N:1

O:4

Dispersal Table:

from to within

A 2.00 1.00 1

B 8.33 1.00 11

C 8.00 4.00 6

D 5.33 2.00 4

E 0.00 1.00 0

F 0.00 3.00 1

G 2.00 6.00 4

H 0.00 1.00 10

I 0.33 2.00 1

J 0.00 2.00 0

K 0.00 1.00 0

L 0.00 1.00 0

M 6.00 2.00 19

N 0.00 1.00 1

O 1.00 5.00 4

===================

Global Cost:

Global Dispersal: 67

Global Vicariance: 33

Global Extinction: 1
