# Supplementary material for: Phylogenomics of the Andean Tetraploid Clade of the American Amaryllidaceae (Subfamily Amaryllidoideae): Unlocking a Polyploid Generic Radiation Abetted by Continental Geodynamics
Source: Front Plant Sci. 2020 Nov 5;11:582422. doi: 10.3389/fpls.2020.582422 (PMC7674842; doi:10.3389/fpls.2020.582422)
Supplement: Supplementary Figure 9 — Area cladogram generated by Bayarealike +J analysis with BioGeoBears through RASP 4.2, using the best maximum likelihood tree found for the partial plastome supermatrix. Areas at any node were limited to three, and only the most likely was mapped onto the tree. [file Data_Sheet_9.PDF]

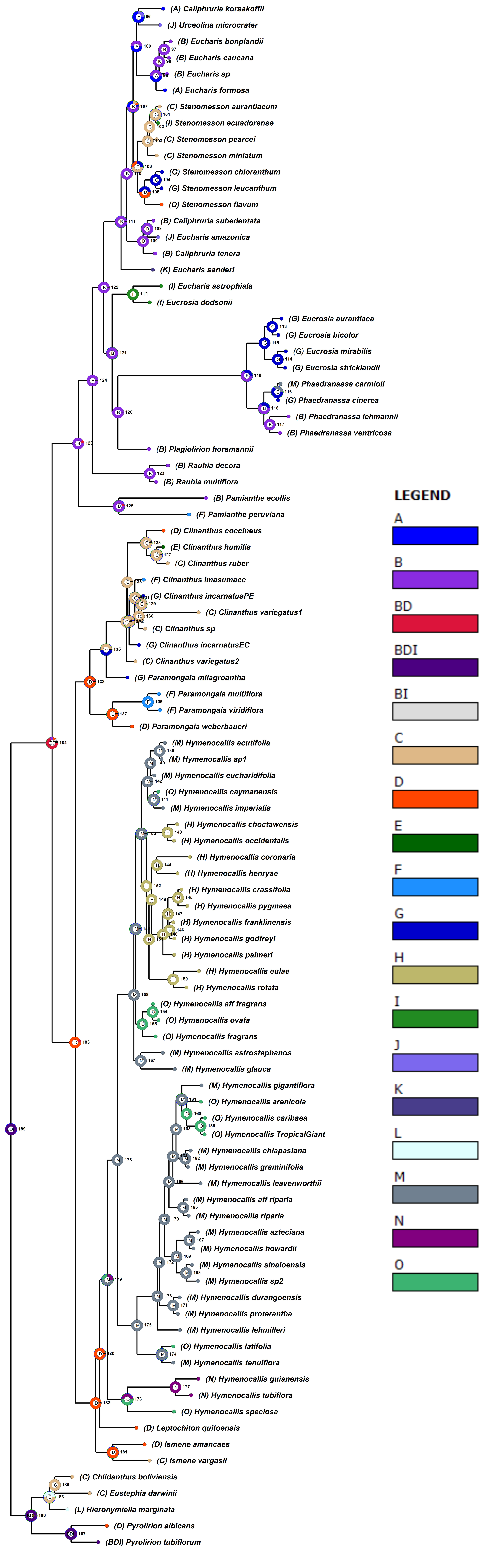
(A) Caliphruria korsakoffii
(J) Urceolina microcrater
(B) Eucharis bonplandii
(B) Eucharis caucana
(B) Eucharis sp
(A) Eucharis formosa
(C) Stenomesson aurantiacum
(I) Stenomesson ecuadorense
(C) Stenomesson pearcei
(C) Stenomesson miniatum
(G) Stenomesson chloranthum
(G) Stenomesson leucanthum
(D) Stenomesson flavum
(B) Caliphruria subedentata
(J) Eucharis amazonica
(B) Caliphruria tenera
(K) Eucharis sanderi
(I) Eucharis astrophiala
(I) Eucrosia dodsonii
(G) Eucrosia aurantiaca
(G) Eucrosia bicolor
(G) Eucrosia mirabilis
(G) Eucrosia stricklandii
(M) Phaedranassa carmioli
(G) Phaedranassa cinerea
(B) Phaedranassa lehmannii
(B) Phaedranassa ventricosa
(B) Plagiolirion horsmannii
(B) Rauhia decora
(B) Rauhia multiflora
(B) Pamianthe ecollis
(F) Pamianthe peruviana
(D) Clinanthus coccineus
(E) Clinanthus humilis
(C) Clinanthus ruber
(F) Clinanthus imasumacc
(G) Clinanthus incarnatusPE
(C) Clinanthus variegatus1
(C) Clinanthus sp
(G) Clinanthus incarnatusEC
(C) Clinanthus variegatus2
(G) Paramongaia milagroantha
(F) Paramongaia multiflora
(F) Paramongaia viridiflora
(D) Paramongaia weberbaueri
(M) Hymenocallis acutifolia
(M) Hymenocallis sp1
(M) Hymenocallis eucharidifolia
(O) Hymenocallis caymanensis
(M) Hymenocallis imperialis
(H) Hymenocallis choctawensis
(H) Hymenocallis occidentalis
(H) Hymenocallis coronaria
(H) Hymenocallis henryae
(H) Hymenocallis crassifolia
(H) Hymenocallis pygmaea
(H) Hymenocallis franklinensis
(H) Hymenocallis godfreyi
(H) Hymenocallis palmeri
(H) Hymenocallis eulae
(H) Hymenocallis rotata
(O) Hymenocallis aff fragrans
(O) Hymenocallis ovata
(O) Hymenocallis fragrans
(M) Hymenocallis astrostephanos
(M) Hymenocallis glauca
(M) Hymenocallis gigantiflora
(O) Hymenocallis arenicola
(O) Hymenocallis caribaea
(O) Hymenocallis TropicalGiant
(M) Hymenocallis chiapasiana
(M) Hymenocallis graminifolia
(M) Hymenocallis leavenworthii
(M) Hymenocallis aff riparia
(M) Hymenocallis riparia
(M) Hymenocallis azteciana
(M) Hymenocallis howardii
(M) Hymenocallis sinaloensis
(M) Hymenocallis sp2
(M) Hymenocallis durangoensis
(M) Hymenocallis proterantha
(M) Hymenocallis lehmilleri
(O) Hymenocallis latifolia
(M) Hymenocallis tenuiflora
(N) Hymenocallis guianensis
(N) Hymenocallis tubiflora
(O) Hymenocallis speciosa
(D) Leptochiton quitoensis
(D) Ismene amancaes
(C) Ismene vargasii
(C) Chlidanthus boliviensis
(C) Eustephia darwinii
(L) Hieronymiella marginata
(D) Pyrolirion albicans
(BDI) Pyrolirion tubiflorum
LEGEND
A
B
BD
BDI
BI
C
D
E
F
G
H
I
J
K
L
M
N
O
